# Quorum sensing in *Vibrio* controls carbon metabolism to optimize growth in changing environmental conditions

**DOI:** 10.1371/journal.pbio.3002891

**Published:** 2024-11-11

**Authors:** Chelsea A. Simpson, Zach R. Celentano, Nicholas W. Haas, James B. McKinlay, Carey D. Nadell, Julia C. van Kessel

**Affiliations:** 1 Department of Biology, Indiana University, Bloomington, Indiana, United States of America; 2 Department of Biological Sciences, Dartmouth College, Hanover, New Hampshire, United States of America; Fred Hutchinson Cancer Research Center, UNITED STATES OF AMERICA

## Abstract

Bacteria sense population density via the cell–cell communication system called quorum sensing (QS). The evolution of QS and its maintenance or loss in mixed bacterial communities is highly relevant to understanding how cell–cell signaling impacts bacterial fitness and competition, particularly under varying environmental conditions such as nutrient availability. We uncovered a phenomenon in which *Vibrio* cells grown in minimal medium optimize expression of the methionine and tetrahydrofolate (THF) synthesis genes via QS. Strains that are genetically “locked” at high cell density grow slowly in minimal glucose media and suppressor mutants accumulate via inactivating mutations in *metF* (methylenetetrahydrofolate reductase) and *luxR* (the master QS transcriptional regulator). In mixed cultures, QS mutant strains initially coexist with wild-type, but as glucose is depleted, wild-type outcompetes the QS mutants. Thus, QS regulation of methionine/THF synthesis is a fitness benefit that links nutrient availability and cell density, preventing accumulation of QS-defective mutants.

## Abstract 2

Vibrio bacteria sense their surroundings, such as changes in cell density and nutrients, and respond to them using various signaling pathways. This study reveals a link between quorum sensing and metabolic regulation that confers fitness advantages in the presence of certain nutrients.

## Introduction

**Fig 1:**
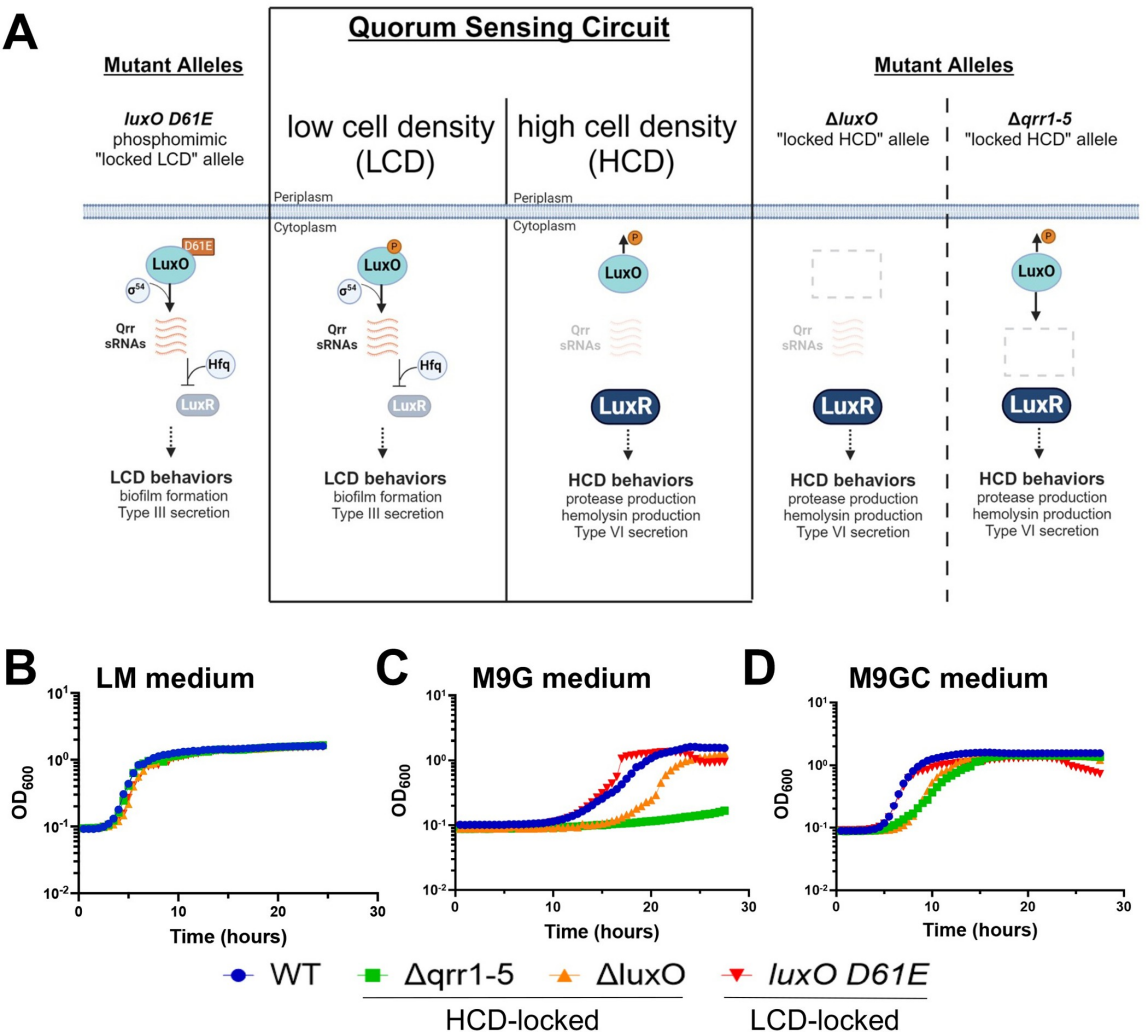
Growth trends of *V*. *campbellii* quorum sensing mutants differ in the absence of amino acids. (A) *V*. *campbellii* QS system and mutant alleles. At LCD, LuxO is phosphorylated and along with Sigma-54 activates transcription of the Qrr small RNAs. The Qrrs together with Hfq repress LuxR production. At HCD, unphosphorylated LuxO is unable to activate transcription of the Qrr small RNAs, allowing for translation of LuxR and production of proteases. The *luxO D61E* allele produces a locked-LCD phenotype. Deletion of *luxO* or the *qrrs* results in phenotypically HCD-locked strains. Created with BioRender.com. (B–D) Growth curves for *V*. *campbellii* DS40M4 strains in LM (B), M9G (C), or M9GC (D) media. The y-axis represents cell density OD_600._ For all panels, the data are from a single experiment that is representative of at least 3 independent biological experiments for each strain under every condition. The data underlying this figure can be found in S1 Data. HCD, high cell density; LCD, low cell density; LM, lysogeny broth marine; QS, quorum sensing; WT, wild-type.

Bacteria monitor their surroundings and respond to changes in cell density through quorum sensing (QS), allowing them to adapt to environmental cues that affect bacterial cell growth and survival [1,2]. Using molecules called autoinducers, cells sense density increases and respond with broad changes in gene expression that alter their behaviors and optimize growth, colonization, infection, and many other processes. QS signaling mechanisms have been studied in several *Vibrio* species [3–6], which are a group of ecologically and economically relevant pathogens of fish, shellfish, and mammals, including humans. Early foundational QS studies were performed with *V*. *campbellii* strain ATCC BAA-1116 (also called BB120), a *V*. *campbellii* isolate that was previously classified as *Vibrio harveyi* [7]. We recently published a comparative study examining the QS signaling and gene regulation in a wild isolate of *V*. *campbellii* DS40M4 to its close relative *V*. *campbellii* BB120 [8]. The DS40M4 strain is an environmental isolate from the deep sea and is naturally competent. Thus, it has become a model organism for us to study QS in a relevant strain with high-throughput genetic methods [9]. Although much of the QS circuit functions similarly in these 2 strains, there were differences in signaling and regulation of downstream genes, and the current model for DS40M4 QS signaling is depicted in S1 Fig [8]. Cells detect and respond to autoinducer signals via multiple histidine kinases that converge to phosphorylate the response regulator LuxO (Figs 1A and S1). At low cell density (LCD) when LuxO is phosphorylated (LuxO~P), LuxO~P activates transcription of the quorum regulatory small RNAs (Qrrs) that posttranscriptionally activate translation of AphA, the LCD master regulator, and repress translation of LuxR, the high cell density (HCD) master regulator (Fig 1A) [10]. At HCD, LuxO is not phosphorylated and is unable to activate the Qrrs, allowing for high expression of LuxR. LuxR activates and represses genes encoding proteins for various behaviors, including type III and type VI secretion systems, proteases, and biofilms (Fig 1A) [8].

*V*. *campbellii* DS40M4 is completely prototrophic and grows robustly in minimal M9 medium with only the addition of carbon (e.g., glucose). However, we observed that HCD-locked mutant strains of DS40M4 grew poorly in minimal media conditions compared to the wild-type parent strain, and this effect was relieved by addition of amino acids to the medium. Here, we determined the molecular mechanisms that enable QS to regulate methionine and tetrahydrofolate biosynthesis genes as cells transition between LCD and HCD states. Our results underscore the relevance of nutrient acquisition—and how it is influenced by QS regulation—in connection to the evolutionary stability of QS.

## Results

### Carbon source determines QS mutant growth trends

Because QS enables cells to respond to external signals, we sought to examine the impact of nutrients on the growth and signaling of DS40M4 strains. As previously performed in other *Vibrio* strains, we engineered mutants that are genetically locked at LCD or HCD via alleles of *luxO*: the *luxO D61E* strain is a phosphomimic allele that constitutively activates transcription of the Qrrs and thus is a LCD-locked mutant; the Δ*luxO* strain is locked at HCD and does not transcribe the Qrrs (Fig 1A) [8,11]. The *luxO* phosphomimic allele had been previously misannotated in *Vibrio cholerae* as *luxO* D47E with an incorrect start codon [11] and other *Vibrio* species share the same earlier start codon. Thus, herein we refer to the phosphomimic allele as *luxO D61E*. We assayed growth of QS mutant strains in the following conditions that are standards for *Vibrio* growth: (1) rich, undefined Lysogeny Broth Marine medium (LM; LB with 2% NaCl); (2) M9 minimal medium with 20 mM glucose (M9G); and (3) M9G with casamino acids (0.2%; M9GC). In this assay, the cells were washed out of LM rich medium from the overnight culture and diluted 1:100,000 into fresh medium (LM, M9G, or M9GC). Growth trends for all strains were similar in LM (Fig 1B), and as previously observed, DS40M4 had a faster growth rate than BB120 in LM (Figs 1B and S2A) [8]. The 2 HCD-locked DS40M4 strains, Δ*luxO* and Δ*qrr1-5*, did not grow well in M9G, with long lag phases that were markedly recovered by the addition of casamino acids (Fig 1C and 1D). Of note, the growth trends were different for BB120: The Δ*qrr1-5* strain exhibited a slower growth rate compared to wild-type, but the Δ*luxO* did not and had a slightly higher growth yield (S2A–S2C Fig). We observed variability in the length of the lag phases for DS40M4 in the Δ*luxO* and Δ*qrr1-5* strains from experiment to experiment (S2D Fig), suggesting that the length of the lag phase was dictated by the presence and abundance of suppressor mutants in the initial inoculum. However, in every case, the Δ*qrr1-5* strain grew the slowest in M9G (Figs 1C, S2B, and S2D), possibly because this strain completely lacks the *qrr* genes, whereas the Δ*luxO* strain encodes these genes but does not activate their expression. Conversely, in both the DS40M4 and BB120 isolates, the phosphomimic *luxO D61E* strain grew robustly in M9G (Figs 1C and S2B). We noted a consistent drop in optical density (OD) in late stationary phase in both M9G and M9GC for the *luxO D61E* strains that did not correlate to a decrease in cell viability (Figs 1C, 1D, S2E, and S2F). To determine if the growth defect was linked to glucose only, we assayed other sugars and observed similar growth phenotypes when DS40M4 strains were grown in either M9G or M9 with added maltose (S2G Fig). We also noted that even in the presence of casamino acids, the 2 HCD-locked strains (Δ*qrr1-5* and Δ*luxO*) had a longer lag phase and slightly decreased growth rate compared to wild-type and *luxOD61E* (Fig 1D), suggesting that there is a further growth defect linked to the HCD-state beyond the carbon source.

*V*. *cholerae* HCD-locked Δ*luxO* has been previously shown to exhibit growth defects in minimal media that are restored in LB medium [12]. In order to determine the general conservation of the growth defects we observed in *V*. *campbellii*, we assayed both *V*. *cholerae* and *V*. *coralliilyticus* Δ*luxO* strains grown in M9G medium. The *V*. *coralliilyticus* strain showed a growth defect similar to DS40M4 with a longer lag phase and slower growth rate (S2H Fig). Although *V*. *cholerae* did exhibit a growth lag in every assay, it was less pronounced than *V*. *coralliilyticus* or *V*. *campbellii* DS40M4 (S2I Fig). Similarly to DS40M4, addition of casamino acids increased growth rates and decreased lag phases for both the *V*. *cholerae* and *V*. *coralliilyticus* Δ*luxO* strains and were similar in growth to wild-type (S2J and S2K Fig). From these data, we conclude that, in the absence of amino acids, the growth rate of multiple *Vibrio* species is dependent on the QS state, and this is mostly alleviated when amino acids are present in the medium.

### Deletion of *metF* improves growth trends of HCD strains

**Fig 2:**
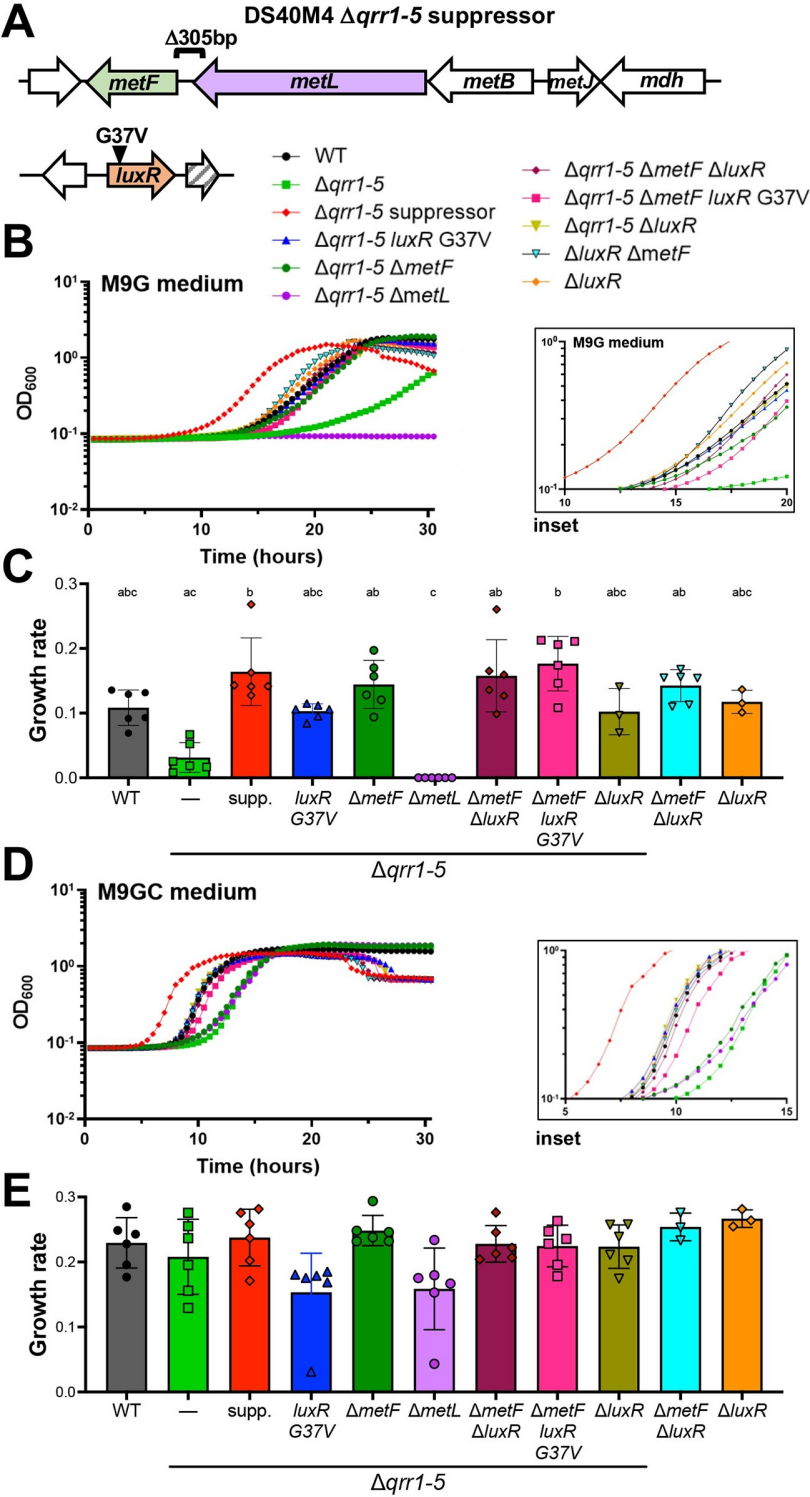
Mutation of *metF* or *luxR* restores growth to HCD-locked strains. (A) Mutations identified in the Δ*qrr1-5* suppressor mutant strain. (B) Culture growth of *V*. *campbellii* DS40M4 strains in M9G medium. (C) Growth rates of strains from panel B are plotted. (D) Growth curves of *V*. *campbellii* DS40M4 strains in M9GC medium. The inset in panels B and D are zoomed-in views of the same data to enable visualization of strains at early time points. (E) Growth rates of strains from panel D are plotted. For panels B and D, the data are from a single experiment that is representative of at least 3 independent biological experiments for each strain under every condition and the y-axis represents cell density OD_600._ For panels C and E, error bars show the mean and standard deviation of at least 3 biological replicates. The slope of the line was derived from the data in exponential phase with at least 5 data points per growth curve. Statistical analyses were performed using the Kruskal–Wallis test (nonparametric) followed by Dunn’s multiple comparisons test (*n =* at least *3*). Different letters above the bars indicate that the pair-wise comparison is significantly different; the same letters above 2 bars indicate no significant differences (*p <* 0.05). There were no significant differences in panel D. The data underlying this figure can be found in S2 Data. HCD, high cell density; WT, wild-type.

To identify a possible genetic link to the limited growth phenotype of the HCD-locked strains, we collected DS40M4 suppressor mutants that grew better in M9G medium than the HCD-locked parent strains Δ*qrr1-5* or Δ*luxO*. Cultures were passaged starting from single isolated colonies grown in liquid M9G medium repeatedly until cultures were turbid (12 passages for the Δ*qrr1-5* strain and 17 passages for the Δ*luxO* strain). Whole genome sequencing of the 2 fastest growing suppressor mutant strains identified mutations in the same 2 loci for both suppressor strains: (1) a 305-bp deletion between genes annotated as *metL* and *metF* and a point mutation resulting in a LuxR missense allele, G37V (the Δ*qrr1-5* suppressor strain; Fig 2A); and (2) a 25-bp deletion in *metF* and a frameshift in LuxR (the Δ*luxO* suppressor strain; S3A Fig). To determine the individual effects of these mutations in an isogenic background strain, we introduced the following mutations in the DS40M4 Δ*qrr1-5* strain: Δ*metF*, Δ*metL*, *luxR* G37V, and/or Δ*luxR*. The Δ*qrr1-5* Δ*metL* strain did not grow in M9G medium and cfu counts remained the same until a decrease was observed at 24 h (Figs 2B and S3F), whereas the Δ*qrr1-5* Δ*metF* strain had an increased growth rate compared to Δ*qrr1-5* (Fig 2B and 2C). The same phenotypes occurred with the Δ*luxO* Δ*metL* and Δ*luxO* Δ*metF* strains, respectively (S3B Fig). The *luxR* G37V allele also increased growth rates compared to the Δ*qrr1-5* parent strain (Fig 2B and 2C) and the Δ*luxO* parent strain (S3B Fig). The combination of both *luxR* and *metF* deletions resulted in growth rates equal to that of the Δ*qrr1-5* suppressor strain (Fig 2C). We also observed similar growth rates with either the Δ*luxR* or *luxR* G37V allele in the Δ*qrr1-5* parent (Fig 2C). Complementation of *luxR* or *metF* in their respective deletion backgrounds resulted in poorer growth, indicating that deletion of *luxR* or *metF* are indeed sufficient to increase growth in the Δ*qrr1-*5 background (S3D Fig). Further, deletion of *metF* also increased growth in the wild-type background (S3E Fig). Addition of casamino acids resulted in much faster growth rates and shorter lag phases for all strains (Figs 2D and S3C), and there were no significantly different growth rates between any strains in casamino acids-containing medium (Fig 2E). We note that 3 strains (Δ*qrr1-5*, Δ*qrr1-5* Δ*metF*, and Δ*qrr1-5* Δ*metL*) had a longer lag phase than all the other strains even in the presence of casamino acids (Fig 2D), similar to what was observed in Fig 1D. These results suggest that HCD-locked strains expressing wild-type *luxR* may have additional growth defects under these conditions. From these data, we conclude that deletion of *metF* and *luxR* in the Δ*qrr1-5* strain increases growth in minimal medium, and growth defects of the HCD-locked strains are rescued by addition of casamino acids.

### The LuxR G37V allele has decreased regulatory function due to decreased DNA binding activity

**Fig 3:**
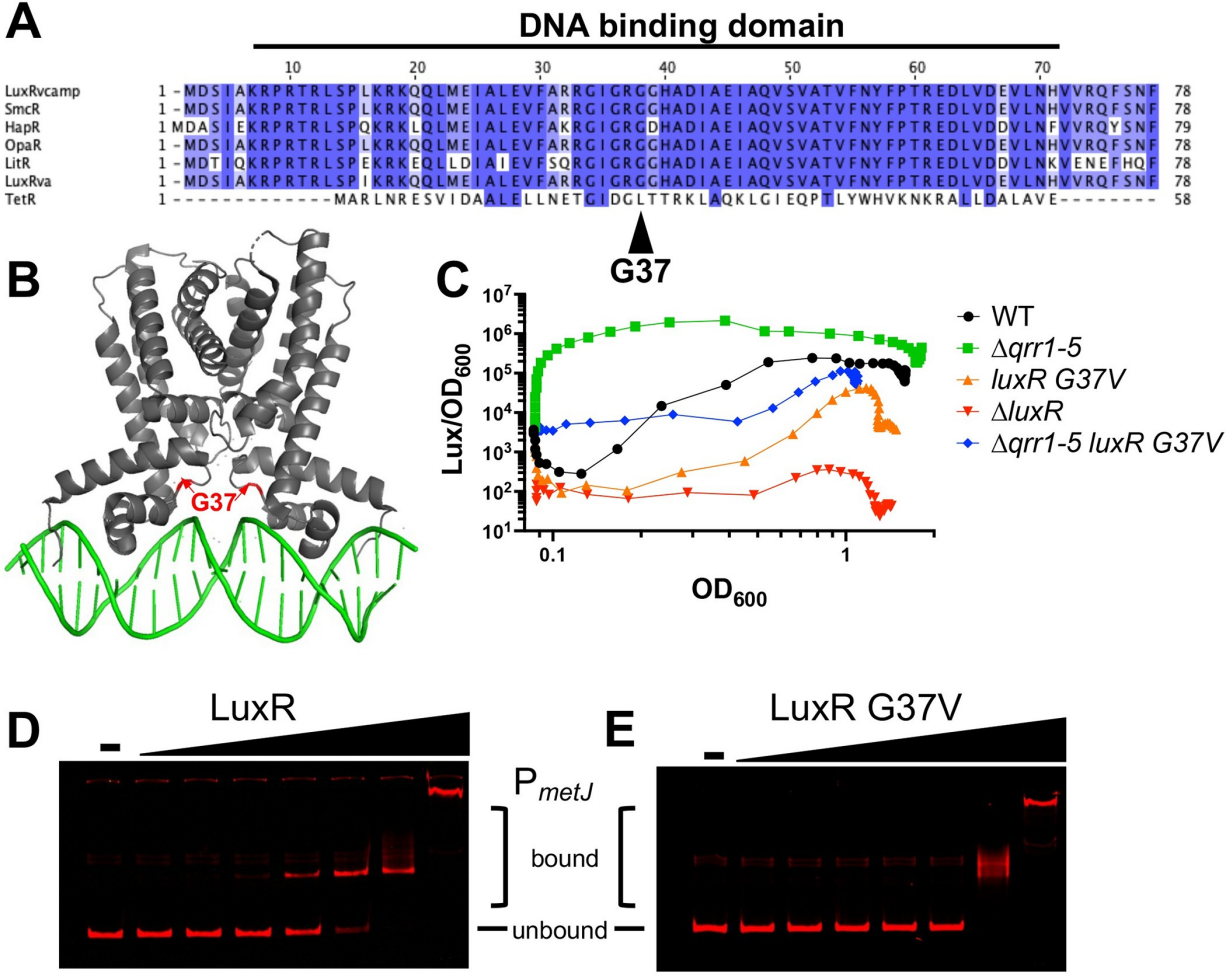
LuxR G37V has decreased DNA-binding activity. (A) The protein sequences for LuxR_Vcamp_ (*V*. *campbellii;* AAA27539), SmcR (*V*. *vulnificus;* AAF72582), HapR (*V*. *cholerae*; ABD24298), OpaR (*V*. *parahaemolyticus;* NP_798895), LitR (*V*. *fischeri;* YP_205560), and LuxR_Va_ (*V*. *alginolyticus;* PDB: 7AMN) aligned to *E*. *coli* TetR (P0ACT4) were aligned using Clustal Omega [19] and the diagram generated using Jalview 2.0 [57]. (B) Structure of LuxR from *V*. *alginolyticus* bound to a repressed DNA sequence [14]. (C) Bioluminescence production (light divided by OD_600_) throughout a growth curve for DS40M4 strains. The data shown are from a single experiment that is representative of at least 3 independent biological experiments for each strain under every condition. (D, E) EMSAs with either purified WT LuxR (D) or G37V LuxR (E) with 5′ IR700 Dye (Integrated DNA Technologies) DNA substrate P*metJ* region (ZC011 and ZC012). Protein concentrations are 0.0005, 0.005, 0.05, .5, 5, 50, and 500 nM, compared to no protein control (“-”). The data underlying this figure can be found in S2 Data and S1 Raw Images. EMSA, electrophoretic mobility shift assay; WT, wild-type.

To determine the mechanism of LuxR in regulation of methionine biosynthesis genes, we examined the G37V missense allele that was isolated in the M9G Δ*qrr1-5* suppressor mutant. The G37 residue is located in the LuxR DNA binding domain and is highly conserved in *Vibrio* LuxR proteins (which are TetR-type proteins) but not in *Escherichia coli* TetR [13] (Fig 3A). This substitution lies in the connecting loop of the helix-turn-helix in *V*. *alginolyticus* LuxR (LuxR_Va_; Fig 3B) [14] and may influence the position of the helix in each monomer and its contact with the DNA. However, we hypothesized that the substitution of a valine for a glycine at this position is subtle and would not drastically alter DNA binding activity, nor does it appear to lie anywhere near the previously identified RNA polymerase interaction domain [13]. To assay the effect of the G37V substitution, we examined bioluminescence production, a direct readout of LuxR’s DNA binding and RNA polymerase-interaction activities [13]. We observed that the introduction of the LuxR G37V allele to either wild-type or Δ*qrr1-5* strains resulted in a ~10-fold decreased light production compared to the parent strain (Fig 3C). This result suggests that the G37V substitution decreased LuxR activity, likely via decreased DNA binding. In *V*. *campbellii* BB120, LuxR binds to the *metJ* promoter and directly represses *metJ* expression in BB120 [15], thus we hypothesized that abrogation of LuxR DNA binding increases growth in M9G by de-repressing *metJ* expression. We purified both the wild-type DS40M4 LuxR and LuxR G37V proteins and assayed DNA-binding activity by electrophoretic mobility shift assays (EMSAs). First, we assayed a well-characterized LuxR DNA-binding site in the *V*. *campbellii* BB120 *luxCDABE* promoter [10,16,17]. We observed that LuxR G37V had a decreased affinity for this DNA substrate compared to wild-type LuxR (S4A Fig). Next, we assayed binding of these 2 proteins to the *metJ* promoter region. We again observed that the LuxR G37V protein bound the P_*metJ*_ region with decreased affinity compared to the wild-type protein (Fig 3D and 3E). No specific binding shifts were observed with either WT or G37V LuxR proteins with a control substrate corresponding to the open-reading frame of *mutS* (S4B Fig), indicating that LuxR had specific DNA binding to *metJ*. We note that both proteins display higher-order shifts at the 500-nM concentration in all gels with all substrates, and we hypothesize that these are due to protein aggregation rather than specific binding, which has been observed with LuxR interactions with larger DNA substrates in other studies [16]. From these collective data, we conclude that LuxR G37V has decreased DNA binding activity both in vivo and in vitro that leads to loss of function at the *metJ* promoter. This aligns with published studies in which substitutions in the analogous residue of the LuxR homolog, HapR, in *V*. *cholerae* led to decreased function [18].

### Quorum sensing regulates transcription of multiple methionine biosynthesis enzyme genes and regulators

**Fig 4:**
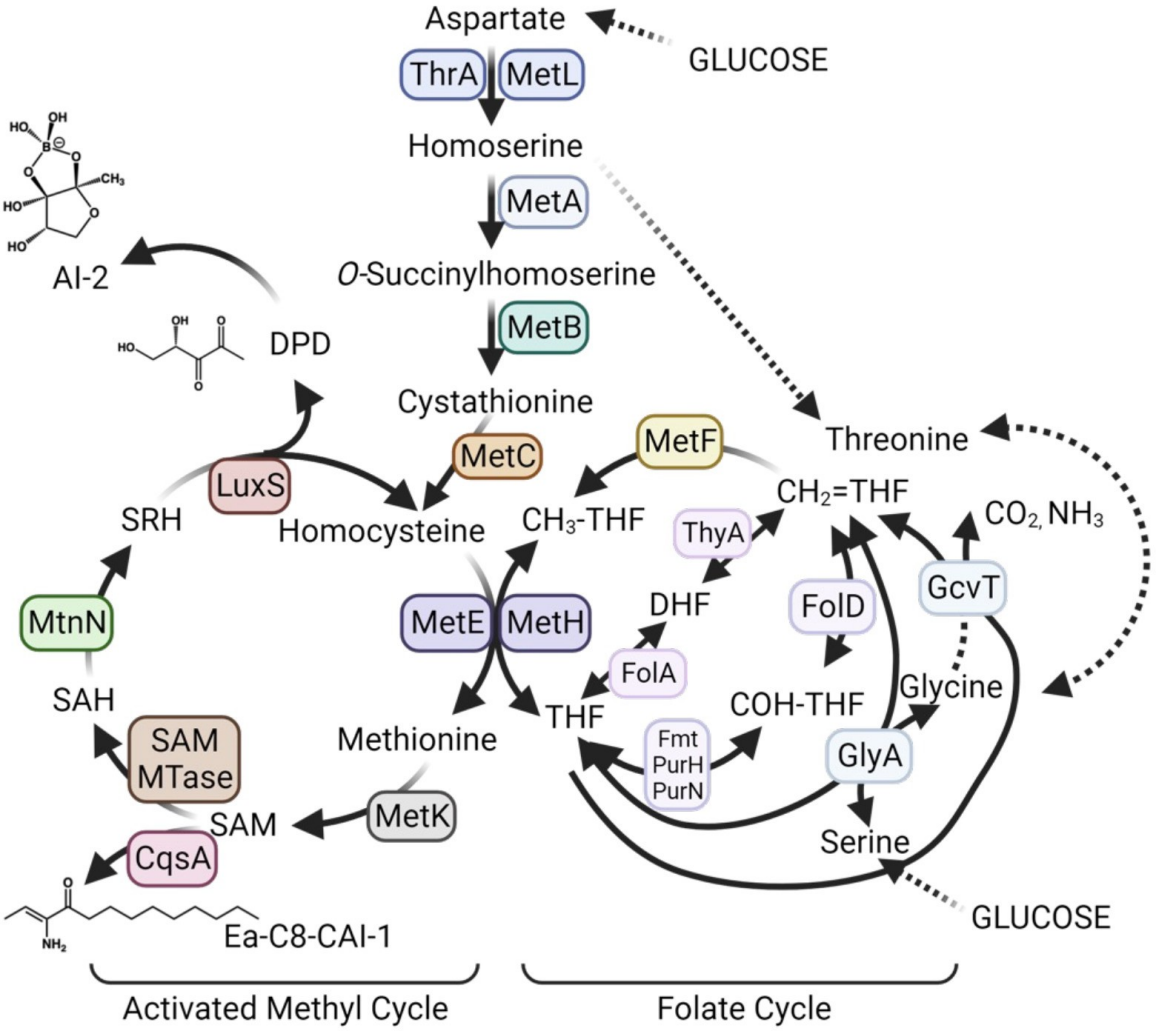
Predicted AMC and folate cycle enzymes in *V*. *campbellii* DS40M4. This is based on *V*. *campbellii* BB120 KEGG gene predictions. Created with BioRender.com.

Biochemical and genetic work in *E*. *coli* and other bacteria has generated a model of conserved key enzymes, substrates, and products in the methionine biosynthesis pathway, including the activated methyl cycle (AMC) and folate cycle. The KEGG [20] database uses this information to predict the presence/absence of biosynthetic pathways present in *V*. *campbellii* BB120 based on gene conservation and ontology. We used this information to diagram the predicted pathways for *V*. *campbellii* DS40M4 (Fig 4). MetF (5,10-methylenetetrahydrofolate reductase) catalyzes the reduction of 5,10-methylenetetrahydrofolate (CH_2_ = THF) to 5-methyltetrahydrofolate (CH_3_-THF), a step in the folate cycle that is the precursor to tetrahydrofolate (THF) synthesis (Fig 4). MetH and MetE are both able to use the CH_3_-THF product and L-homocysteine to produce L-methionine and THF. Methionine is then used as a substrate to produce *S-*adenosyl-L-methionine (SAM), *S-*adenosyl-L-homocysteine (SAH), and *S*-(5-deoxy-D-ribos-5-yl)-L-homocysteine (SRH) [21]. SRH is used by the enzyme LuxS [22] to regenerate L-homocysteine and the byproduct 4,5-dihydroxy-2,3-pentanedione (DPD), the precursor of autoinducer AI-2 [23].

**Fig 5:**
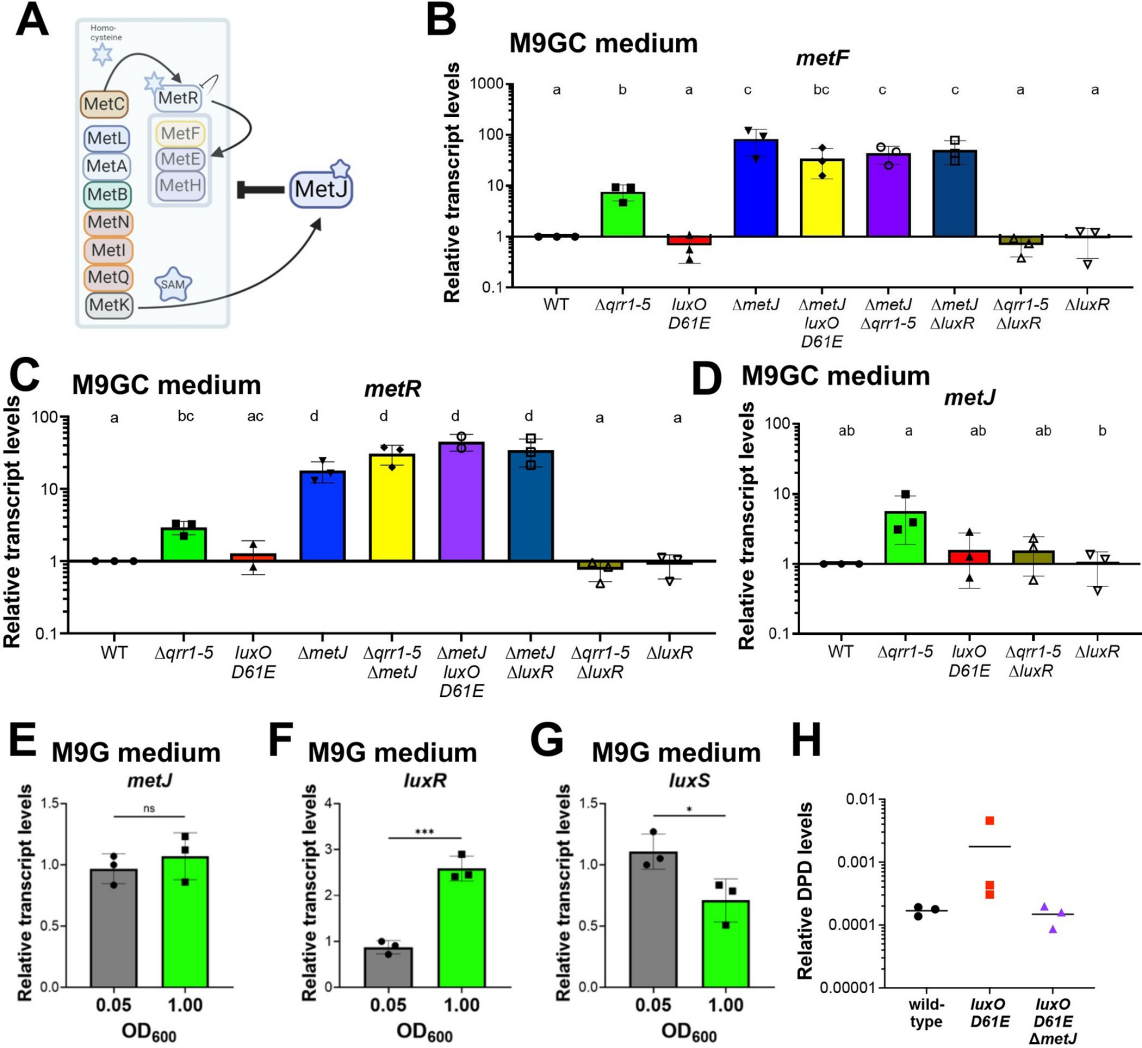
LuxR regulation of methionine biosynthesis genes. (A) Predicted regulation hierarchy of the methionine biosynthesis genes. Created with BioRender.com. (B–G) Relative transcript levels determined by RT-qPCR for cultures grown in M9GC medium (B–D) or M9G medium (E–G). Error bars show the mean and standard deviation of 3 biological replicates. (B–D) Different letters above the bars indicate that the pair-wise comparison is significantly different; the same letters above 2 bars indicate no significant differences (*p <* 0.05). One-way analysis of variance (ANOVA) was performed on log-normalized data (normally distributed, Shapiro–Wilk test; *n* = 3 biological replicates, Tukey’s multiple comparisons test). (E–G) Student’s–test was performed data (normally distributed, Shapiro–Wilk test; *n* = 3 biological replicates; *p* < 0.05, *; *p* < 0.001, ***). (H) Quantification of DPDQ (derivatization of DPD, the precursor to AI-2) via mass spectrometry from supernatants from strains grown in M9G medium and collected at HCD. DPDQ levels were normalized to an internal control (Verapamil) spiked in every sample. The mean of 3 independent biological replicates is shown. The data underlying this figure can be found in S3 Data. HCD, high cell density; WT, wild-type.

In *E*. *coli* and other bacteria, methionine biosynthesis genes are tightly regulated: MetR, activated by homocysteine [24], activates expression of methionine synthesis genes, and MetJ, which itself is activated by SAM [25], opposes MetR regulation [26] (Fig 5A). We hypothesized that locked strains grow poorly in minimal media because these strains are either over- or under-expressing the AMC and/or folate cycle genes at levels different than wild-type in a growth curve. To test this hypothesis, we first determined if MetJ acts as a repressor of *metF* as predicted by *E*. *coli* data. We isolated RNA from cells grown to HCD (OD_600_ = 1.0) in M9GC because not all strains grew in M9G, thus we could not compare each genotype. We observed that *metF* expression was strongly repressed by MetJ, as predicted based on the *E*. *coli* literature [27] (Fig 5B). Further, *metF* expression was significantly activated in the Δ*qrr1-5* strain compared to wild-type, which was dependent on the presence of *luxR* (Fig 5B). Deletion of *metJ* was epistatic to all QS genotypes. Expression of *metR* mirrored that of *metF* in all strain backgrounds (Fig 5C). We noted that the expression level of *metF* in the wild-type background was significantly lower than that of the Δ*qrr1-5* strain, even though the cells were collected at HCD. From these data, we conclude that MetJ acts as a repressor of *metF* and *metR* in *V*. *campbellii*.

We had shown previously (through in vivo ChIP-seq and in vitro biochemical assays) that LuxR binds to the promoter of *metJ* in *V*. *campbellii* BB120 to repress its expression [15] instead suggested that cells locked at HCD (Δ*qrr1-5*) somehow activated *metJ*.

These results all seemed conflicting: MetJ repressed *metF*, but LuxR activated *metJ*. Thus, we postulated that there is a circular regulatory pattern that balances expression of the *met* genes: at HCD, LuxR activates the *metJ* repressor, thus tempering expression of the *met* genes. This tracks with the observation that a wild-type strain expresses approximately 10-fold lower levels of *metF* than a locked HCD Δ*qrr1-5* strain that does not experience shifts from LCD to HCD. To examine levels in a wild-type strain at the actual low density (OD_600_ = 0.05) versus high density (OD_600_ = 1.00), we collected cells grown in M9G and measured transcript levels. We note that we were able to use the M9G medium with wild-type in this experiment, whereas we could not in the previous experiment because not all strains grew in M9G. We observed that *metJ* levels did not change from LCD to HCD, whereas control measurements of *luxR* confirmed that the cells shifted from LCD to HCD (Fig 5E and 5F).

Based on these results, we conclude the following: (1) MetJ represses *metF* and *metR*, and this is epistatic to QS regulation; (2) a HCD-locked Δ*qrr1-5* strain has increased expression of *metJ*, *metF*, and *metR*; and (3) LuxR—either directly or indirectly—activates *metJ*, *metF*, and *metR* expression in the absence of Qrrs.

### DS40M4 growth is optimal with a balance of the activated methyl cycle and folate cycle

**Fig 6:**
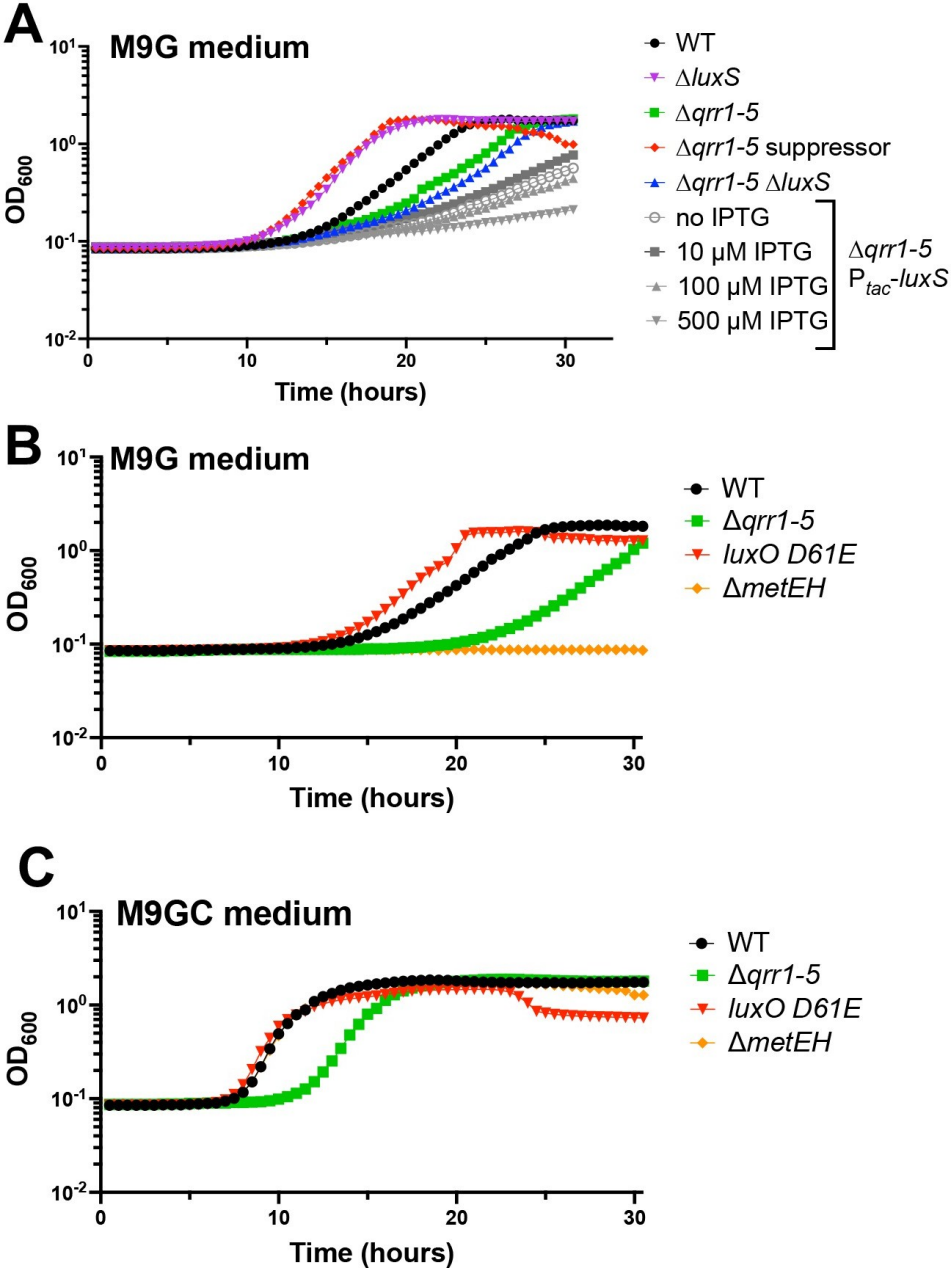
Altering flux between the AMC and folate cycle affects culture growth. (A–C) Growth curves for *V*. *campbellii* DS40M4 strains grown in M9G (A, B) or M9GC (C) medium. The y-axes represent cell density OD_600._ The data shown are from a single experiment that is representative of at least 3 independent biological experiments for each strain under every condition. The data underlying this figure can be found in S4 Data. AMC, activated methyl cycle; WT, wild-type.

Based on our observation that poor growth of HCD-locked strains was alleviated by deletion of *metF*, we hypothesized that the HCD-locked strains produced higher levels of proteins involved in the AMC and/or the folate cycle. Increased MetF enzyme concentrations predictably would decrease the pool of CH_2_ = THF and increase CH_3_-THF pools, possibly affecting the AMC. Thus, we next tested the effect of altering AMC substrate pools via deletion of *luxS*. The Δ*luxS* strain had increased growth in M9G medium, with a short lag phase much like the Δ*qrr1-5* suppressor mutant (Fig 6A). Conversely, deletion of *luxS* in the Δ*qrr1-5* background had only slightly decreased growth compared to the parent and eventually reached a similar density to wild-type (Figs 6A and S3F). Overexpression of *luxS* via an IPTG-inducible promoter in the Δ*qrr1-5* background led to lower growth rates; higher IPTG levels correlated with decreased growth trends (Fig 6A).

Our results underscored the observation that deletion of *metF* increased growth in M9G, whereas deletion of *metL* resulted in no growth in medium without amino acids (Fig 2B). We next assayed whether deletion of *metEH* would be viable in media without amino acids. Our results showed that the Δ*metEH* strain was unable to grow in M9G alone (Fig 6B), supporting the importance of this pathway for growth in M9G medium without amino acids in *V*. *campbellii*. Collectively, these data show that deletion of the *luxS* or *metF* gene increased growth rates, and alteration of these levels in QS-locked strains exacerbated the growth defect. However, deletion of other genes predicted to be important in the folate or AMC cycles resulted in no growth in minimal medium.

Our data thus far suggested that QS regulates some genes in the AMC and/or THF pathways, but some genes are essential in minimal medium and some are not. There is a very clear connection between these 2 pathways and autoinducer synthesis; all known autoinducers in *V*. *campbellii* are synthesized from SAM (Fig 4). Although LuxS is an integral enzyme in the methionine biosynthesis pathway, previous studies have not uncovered regulation of *luxS* by MetJ or LuxR. We next asked how QS and cell density impact autoinducer synthesis, and specifically AI-2. We measured *luxS* transcript levels in wild-type cells grown in M9G at low and high densities, and we observed that *luxS* levels significantly but modestly decrease from LCD to HCD (Fig 5G). Next, we assayed DPD synthesis, the precursor to AI-2 [28], via mass spectrometry (Fig 4). Strains were grown in M9G to HCD (overnight cultures, OD_600_ >1.4) and supernatant extracts were chemically derivatized by adding OPD (ortho-phenylenediamine) which reacts with DPD (4,5-Dihydroxy-2,3-pentanedione) to form DPDQ. We observed that the LCD-locked strain, *luxO D61E*, produced approximately 10-fold more DPD than wild-type, and the *luxO D61E* Δ*metJ* strain produced similar levels to wild-type, although no levels were significantly different (Fig 5H). These results qualitatively correlate with our observation that *luxS* transcript levels are higher at LCD compared to HCD (Fig 5G); however, no definitive conclusion can be made. From these results, we suggest that (1) *luxS* is repressed at HCD; (2) overexpression of *luxS* at HCD caused growth delays; and (3) DPD production decreases at HCD, and this is alleviated by deletion of *metJ*.

### The Qrrs regulate growth of *V*. *campbellii* in minimal medium

**Fig 7:**
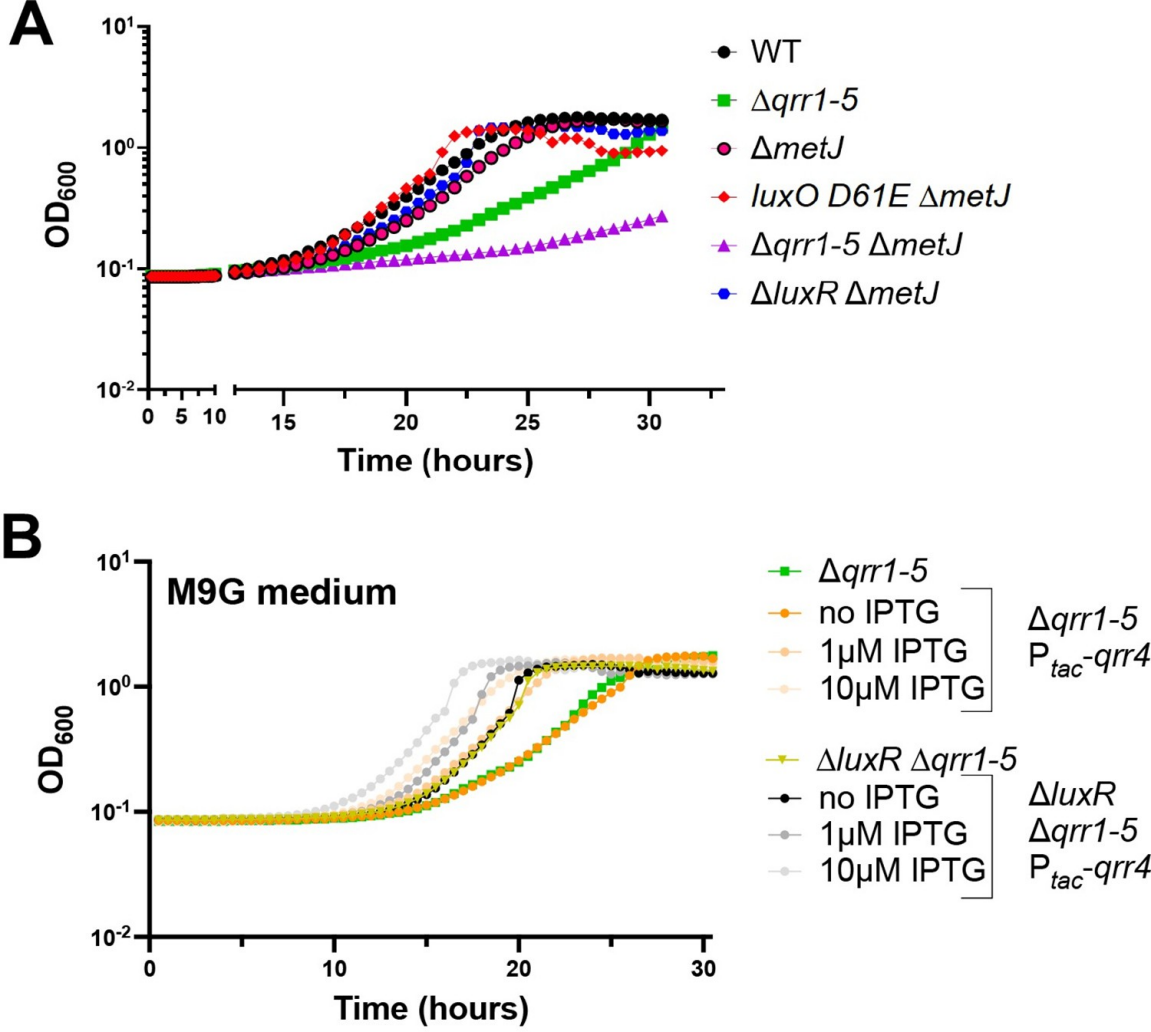
The Qrrs regulate growth of *V*. *campbellii* in minimal medium. (A, B) Growth curves for *V*. *campbellii* DS40M4 strains in M9G medium. The y-axis represents cell density OD_600._ The data shown are from a single experiment that is representative of at least 3 independent biological experiments for each strain under every condition. The data underlying this figure can be found in S4 Data. WT, wild-type.

We next assayed the effect of deleting the MetJ master regulator on growth in minimal media. In the Δ*qrr1-5* background, deletion of *metJ* led to even more severe growth defects when grown in M9G (Fig 7A). We also noted that deletion of *metJ* does not affect growth in strains expressing the Qrrs at LCD (e.g., the wild-type background) (Fig 7A). Thus, we postulated that the Qrrs might also control methionine biosynthesis genes at LCD independently of LuxR through posttranscriptional regulation. To test our hypothesis, we performed growth assays with strains expressing Qrr4 under control of an inducible P_*tac*_ promoter in strain backgrounds with or without *luxR*. We chose to study Qrr4 because multiple studies have shown that *Vibrio* Qrrs have nearly identical functions when expressed from an ectopic promoter [29]. In M9G medium, increased Qrr4 expression improved growth in both the Δ*qrr1-5* strain and in the Δ*qrr1-5* Δ*luxR* strain (Fig 7B). These results indicate that Qrr4 (and possibly all the Qrrs) positively influences growth in glucose minimal media independently of LuxR.

### Wild-type DS40M4 strains outcompete LCD- and HCD-locked mutant strains in limited nutrients

**Fig 8:**
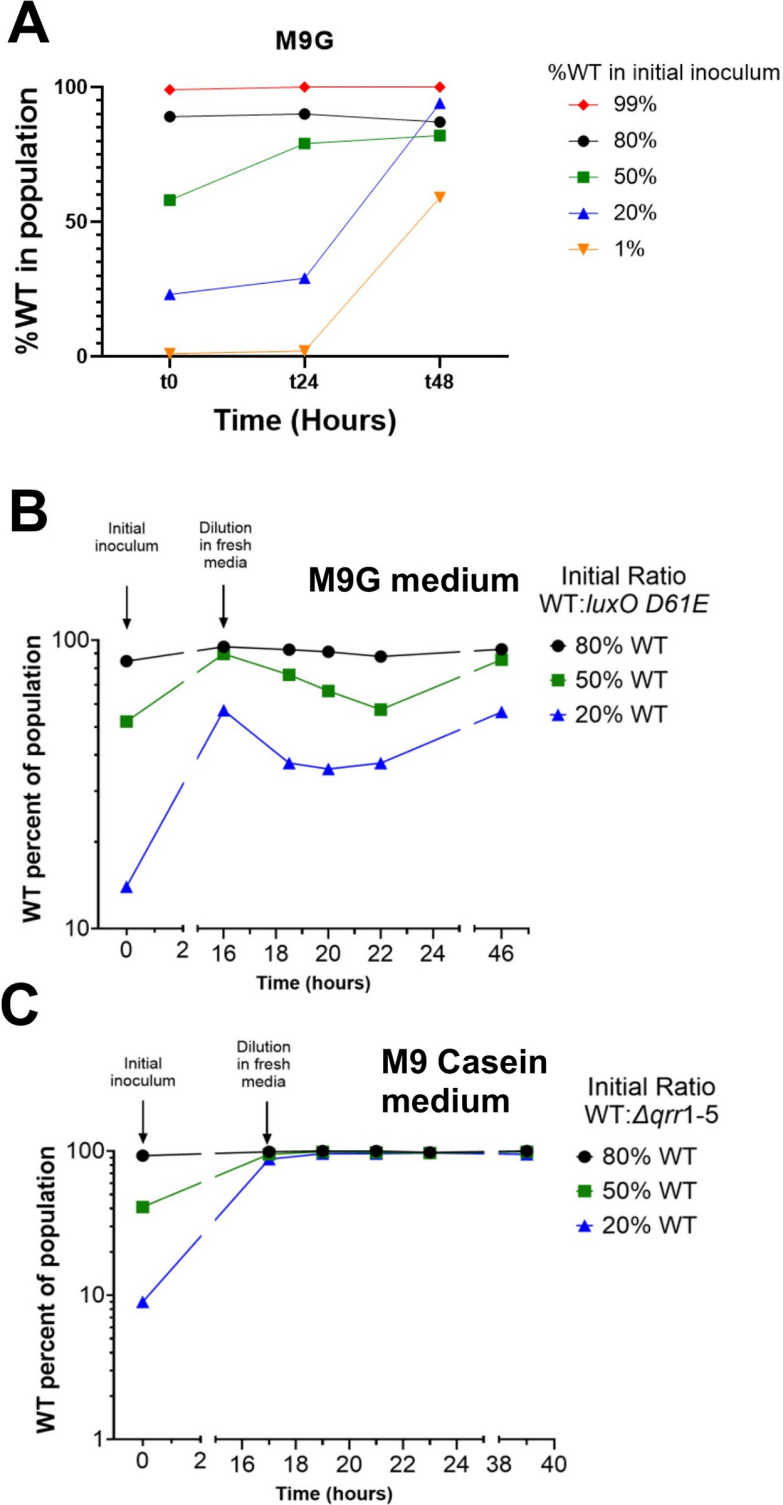
WT cells outcompete locked-LCD cells in co-culture in minimal medium. (A) WT (marked with Tm) and *luxO D61E* (marked with Spec) strains of DS40M4 were inoculated at initial frequencies from 1%–99% WT in M9G medium and allowed to grow for 48 h. (B, C) WT (marked with Tm) and *luxO D61E* (marked with Spec, panel B) or Δ*qrr1-5* (marked with Spec, panel C) strains of DS40M4 were inoculated at initial frequencies of 80%, 50%, or 20% WT in (B) M9G medium or (C) M9 Casein medium and grown to near stationary phase at t = 16 h, and then diluted to maintain growth in log-phase. Colony-forming units (cfu/mL) were measured at each time point using selective antibiotics for each strain. The total population number was calculated, and the data are represented as the percent of WT cells in the total population of WT and *luxO D61E* or WT and Δ*qrr1-5*. The data are representative of 3 biological experiments. The data underlying this figure can be found in S5 Data. cfu, colony-forming unit; LCD, low cell density; WT, wild-type.

Collectively, our observations demonstrated that *V*. *campbellii* DS40M4 strains that are incapable of sensing population density (i.e., LCD-locked or HCD-locked strains) do not regulate methionine biosynthesis or the folate cycle optimally, leading to altered growth trends in M9G (limited nutrient conditions). We hypothesized that QS regulatory control of 2 key pathways—AMC and folate cycle—evolved to optimize growth in poor nutrient conditions. Thus, we assayed the fitness of wild-type DS40M4 compared to our genetically engineered *luxO D61E* (LCD-locked) and the Δ*qrr1-5* (HCD-locked) strains. First, we competed wild-type and the *luxO D61E* strain in M9G batch cultures from a range of initial population frequencies. The wild-type outcompeted the *luxO D61E* strain at every ratio tested (Fig 8A).

Growth on glucose in M9G requires enzymes that are inherently not shared resources. Conversely, growth on substrates such as casein requires production of extracellular proteases to digest the casein into peptides and amino acids that are also utilizable by neighboring cells. In *Vibrio* species including *V*. *campbellii*, production of proteases is often positively controlled by QS [30–32], leading to group production of amino acids that, depending on flow environment, can be shared as “public goods.” Thus, LCD-locked mutants that do not produce proteases cannot grow on their own (S5A Fig), but they can potentially exploit the products of protease-producing cells. In stark contrast to our results competing wild-type and the *luxO D61E* strain in M9G medium, when grown in M9 with casein, the *luxO* D61E strain was able to outcompete wild-type at most ratios (S5B Fig), indicating that wild-type production of proteases was exploited by the LCD-locked mutant, allowing it to thrive in the population. However, when there is a source of readily available amino acids (casamino acids) in the media, the wild-type and LCD-locked mutant population frequencies were maintained, suggesting a similar fitness (S5C Fig). This short-term competition result aligns with several published evolution studies of the factors influencing success or failure of *V*. *campbellii* QS-mutants in mixed cultures [33–35].

We were curious why the LCD-locked *luxO* D61E strain could not outcompete wild-type when co-cultured in M9G, given that the *luxO* D61E strain grew robustly in M9G monocultures. We also hypothesized that because the *luxO* D61E strain grew the fastest that it should consume glucose the fastest. Indeed, quantification of specific glucose consumption rates in M9G monocultures of the QS mutants, using high-pressure liquid chromatography (HPLC) measurements of glucose, showed that the *luxO* D61E strain consumed glucose the fastest per cell, though not significantly faster than the wild-type strain (S6A Fig). All strains consumed all of the 20 mM glucose provided (S6B Fig) (all readings were below detection limits at the end of the time course for all replicates and all strains). Thus, the wild-type advantage cannot be attributed to differences in net glucose utilization.

The above specific consumption rates use values that encompass glucose consumption over entire growth curves. Because the *luxO* D61E strain is LCD-locked, we hypothesized that it might have an advantage at low cell densities in M9G cocultures, whereas the wild-type advantage might come into play towards the end of culture growth. To test this hypothesis, we tracked the wild-type population frequency when cocultured with the *luxO* D61E strain in M9G through serial transfers into fresh M9G at a target initial cell density of OD_600_ = 0.05. Cocultures were transferred in late-exponential phase to keep cocultures in exponential phase. At each dilution, for all 3 initial frequencies tested, the wild-type proportion decreased (Fig 8B). As the cells neared stationary phase again (around the 16-h and 46-h time points), the wild-type proportion increased for all 3 ratio cultures. This pattern was observed in each independent biological experiment (S6C Fig). Although we cannot currently attribute these trends to a specific metabolic advantage (e.g., changes in specific glucose uptake rates at different cell densities), these observations suggest that (1) at LCD when there was abundant fresh carbon (glucose) present, the LCD-locked strain was able to outcompete the wild-type strain; and (2) as the cell density neared stationary phase, wild-type was able to make up for its log-phase losses and dominate the population, which we hypothesize occurred because carbon was nearing depletion. Conversely, when we performed the same experiment with co-cultures of wild-type and Δ*qrr1-5* strains in M9 Casein, the Δ*qrr1-5* strain was nearly completely lost by the first time point of dilution (Fig 8C). Collectively, these results indicate that the type and availability of nutrients drives the frequency of QS-defective strains in co-culture conditions.

## Discussion

The QS field has broadly studied the evolution of QS and its maintenance or loss in bacterial communities and whether cells have evolved to use different nutrient sources that might be present under differing flow conditions [36]. A key focus is the appearance and prevalence of QS null mutants—cells that do not participate in signaling, sensing, and/or responding but still benefit from QS-regulated behaviors occurring at the population level [20]. Here, we show that the growth rate of *V*. *campbellii* DS40M4 in minimal media lacking amino acids is dependent on the QS state. HCD-locked mutants exhibited poor growth in minimal media with only glucose as the carbon source, which was alleviated in suppressor mutants. Our data suggest that HCD-locked mutants grow poorly under these conditions because they express genes involved in the folate cycle at high levels, even at LCD and lag-phase.

**Fig 9:**
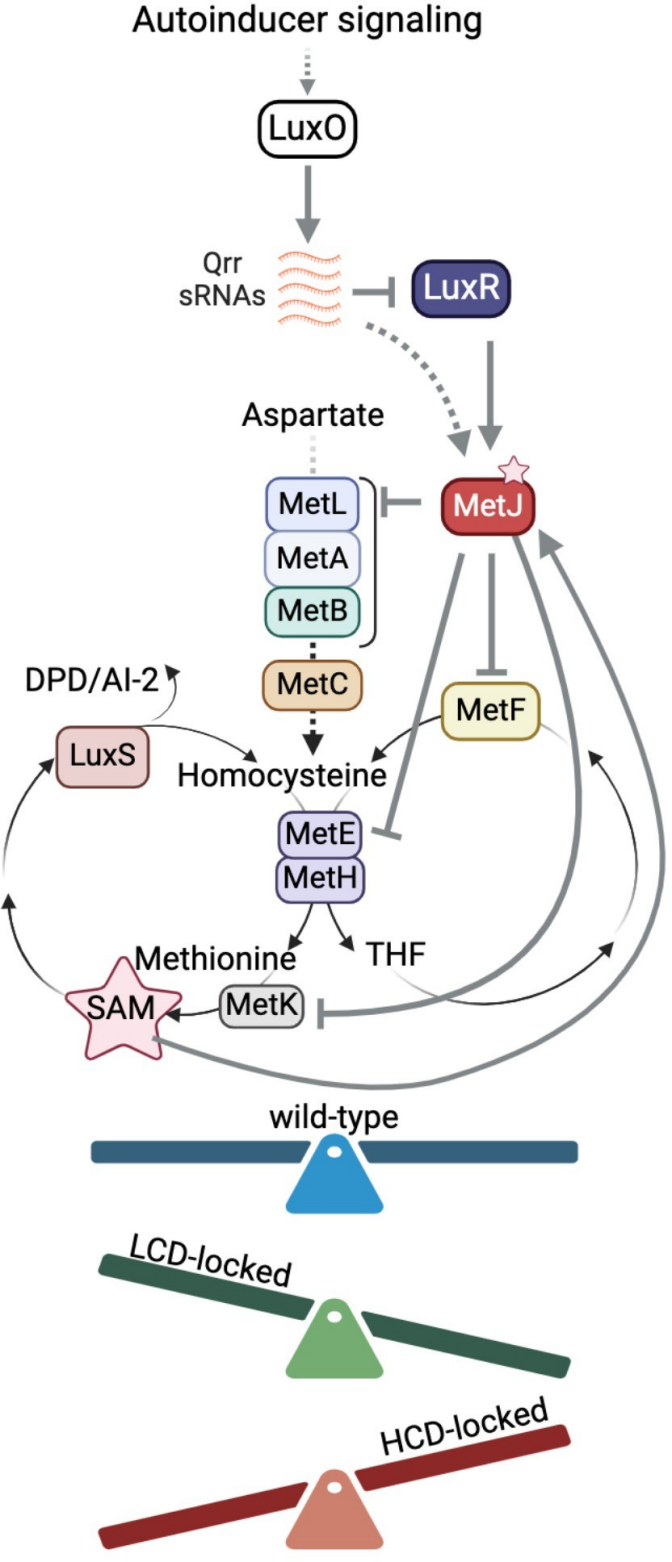
Model of QS regulation of methionine flux in M9G. Wild-type *V*. *campbellii* switches between cell density states during growth, and regulatory feedback loops promote or repress expression of genes in the AMC and folate pathways, resulting in a balanced flux of both systems. Strains locked at LCD or HCD cannot oscillate between states, and thus have growth defects under nutrient-limited conditions. Created with BioRender.com. AMC, activated methyl cycle; HCD, high cell density; LCD, low cell density; QS, quorum sensing.

The 2 suppressor mutants isolated were instrumental in revealing the regulatory network connected by LuxR, the Qrrs, and the AMC and folate pathways. We propose a model in which wild-type cells that can switch between cell density states have the ability to balance—through feedback regulation—expression of the AMC and folate pathways. Conversely, strains locked at LCD are skewed toward the AMC pathway, and strains locked at HCD are skewed toward the folate pathway (Fig 9). Our data show that LuxR activates *metJ*, and MetJ represses several *met* genes. Although we have not uncovered every regulatory mechanism nor feedback loop, we propose that QS regulates the flux between the AMC and folate pathways to optimize growth in medium without amino acids added. Such regulatory connections between QS and various metabolic processes are beginning to unfold in other systems [37,38]. In *V*. *cholerae*, the Qrrs alter the flux of pyruvate metabolism by repressing the translation of AlsS, which is needed to convert fermentable carbon sources into neutral products avoiding acidosis [39]. In *Burkholderia* species, methionine biosynthesis genes (e.g., *metE*, *metF*, and *ahcY*) are activated by QS signals [40]. Thus, our study provides a new basis for studying QS regulation of glucose catabolism.

Broadly, we postulate that this *V*. *campbellii* DS40M4 strain acquired a LuxR binding site in the *metJ* promoter that benefitted the mutant, perhaps under limited nutrient conditions, which has been maintained over time. Metabolism regulation via QS could benefit *V*. *campbellii* in its multiple native environments. As a planktonic free-living organism, *V*. *campbellii* likely has limited nutrient sources aside from complex carbohydrates (e.g., chitin) available in marine ecosystems. However, during host infection (e.g., shrimp, fish), *V*. *campbellii* likely has access to a plethora of nutrients, certainly including digestible proteins. In limited nutrient environments, we hypothesize that QS enables *V*. *campbellii* to optimize flux of essential biosynthesis pathways, outcompete possible competitors (QS mutants), and sustain growth. However, in nutrient-rich environments, such as the host, perhaps competition with QS mutants or other microbes may impact virulence, thus requiring cells to couple metabolism with production of public goods. Indeed, in a mouse infection model of *Vibrio cholerae*, mutant strains with deletions in either *metJ*, *metR*, or *glyA* had severe colonization defects [41], underscoring the importance of nutrient utilization and flux regulation in different environments.

In long-term evolution studies, *V*. *campbellii* BB120 optimizes growth under conditions where public goods (proteases) are required for nutrient acquisition (casein); mutants that either do not cooperate or constitutively produce public goods are less fit than wild-type in long-term serially bottle-necked experiments [33]. In addition, populations that disperse through motility, escape QS mutants and maintain cooperation [35]. In *V*. *campbellii* DS40M4, LCD-locked cells maintained the advantage and outcompeted wild-type when grown in minimal media supplemented with a source of fresh glucose. However, as the nutrients in the environment were depleted, wild-type cells outcompeted these LCD-locked cells. As a note, these strains were back-diluted 1:100,000 when mixed at the desired ratios. Upon back-dilution, we hypothesize that the wild-type strain does not automatically revert to the LCD state. The switch from HCD to LCD requires multiple changes, minimally the following switches in the QS system: (1) loss of binding of autoinducers to receptors; (2) phosphorylation of LuxU/LuxO; (3) synthesis of the Qrr sRNAs, synthesis of AphA; and (4) turnover of LuxR protein, either by regulated degradation [42] or by dilution through cell doubling. This process has been shown to take multiple cell divisions in which QS-regulated behaviors are turned off (e.g., bioluminescence) over time [29]. Thus, upon initial mixing, the wild-type strains continue the HCD “programming” for some time. We hypothesize that this is possibly why LCD-locked cells are able to outcompete the wild-type cells immediately after back-dilution. We propose that because LCD-locked cells are incapable of QS and thus unable to alter flux of the AMC/folate pathways, they are ultimately unable to outcompete wild-type cells. Conversely, in media with casein—requiring extracellular digestions with secreted proteases—LCD-locked cells were able to outcompete wild-type unless the frequency of wild-type in the population was very low. In this ratio, wild-type likely produced a limiting amount of digested casein that barely, if at all, supported growth of itself and the LCD-locked strain.

Cooperative behaviors are coordinated by QS, yet they are inherently subject to QS mutants that exploit public goods production. In several bacterial model systems, QS null mutants are isolated in wild environments; for example, in *Pseudomonas aeruginosa*, mutants lacking LasR are often isolated from infected patients [43]. While *hapR-*null *V*. *cholerae* mutants have also been isolated from water samples and patients in China [44], it is yet unclear if wild populations of *V*. *campbellii* or other more closely related *Vibrio* species consist of QS mutants. *Vibrio* QS signals are produced by AMC enzymes and substrates, including LuxS/SRH, LuxM/SAM, and CqsA/SAM [45–47]; methionine is converted to SAM, which is ultimately converted into autoinducers. In *V*. *campbellii* BB120, autoinducer concentrations increase as cell density increases [48]. However, increased production of AMC and folate cycle enzymes may also be a direct benefit as cell density increases and nutrients are depleted. Thus, we propose that *V*. *campbellii* is an example of a QS system that regulates private goods (methionine and THF) as both a direct benefit and because the key metabolite involved is itself inherently connected to QS signal production.

We also observed that the relative advantage or disadvantage of the *luxO D61E* mutation is highly dependent on prevailing nutrient conditions. Though QS null mutants can exploit wild-type in certain niches, QS is maintained overall in this instance because the ability to adjust to different environmental stimuli outweighs the cost of being occasionally exploited. Future studies of the genetic composition of planktonic and host-colonized *Vibrio* strains will provide important context for the evolution and maintenance of QS in *Vibrios* and other bacteria.

## Experimental procedures

### Bacterial strains and media

*V*. *campbellii* and *V*. *coralliilyticus* strains (S1 Table) were grown at 30°C on lysogeny broth marine (LM) medium (lysogeny broth (LB) supplemented with an additional 10 g NaCl L^-1^) or in M9 minimal media (1× M9 salts, 2 mM MgSO_4_, 0.1 mM CaCl_2_, 3% NaCl) supplemented with 20 mM glucose, 0.5% casein, and 0.2% casamino acids. *E*. *coli* and *V*. *cholerae* strains (S1 Table) were grown in LB or in M9 minimal media (1× M9 salts, 2 mM MgSO_4_, 0.1 mM CaCl_2_, 1% NaCl) supplemented with 20 mM glucose or 0.2% casamino acids at 37°C. Where appropriate, *Vibrio* and *E*. *coli* strains were supplemented with kanamycin (100 μg ml^-1^), spectinomycin (200 μg ml^-1^), gentamicin (100 μg ml^-1^), or trimethoprim (10 μg ml^-1^). Plasmids were transferred from *E*. *coli S17-1λpir* to *Vibrio* strains by conjugation on LB plates. Exconjugants were selected on LM plates with polymyxin B at 50 U ml^-1^ and the appropriate selective antibiotic.

### Strain construction

Chitin-independent transformations were performed in DS40M4 according to Simpson and colleagues [9]. Strain construction details are available upon request. All genome or plasmid genotypes were confirmed by Sanger sequencing.

### Growth curves

Overnight cultures were grown in LM in culture tubes, 500 μl of cells were spun down, resuspended in 500 μl M9 medium, spun down again, and resuspended in 500 μl M9 then diluted 1:100,000 in 200 μl media. OD_600_ was measured every 30 min for 30 h using the BioTek Cytation 3 Plate Reader or BioTek Synergy H1 Plate Reader (temperature: 30°C for *V*. *campbellii* and *V*. *coralliilyticus*; 37°C for *V*. *cholerae*).

### Bioluminescence assays

For the bioluminescence growth assays, overnight cultures were grown in LM, washed with M9G medium, and back-diluted 1:100,000 in 200 μl media with selective antibiotics as needed in a black-welled clear-bottom 96-well plate (all adjacent wells left empty to avoid light carryover). OD_600_ and/or bioluminescence were measured every 30 min for 22 to 30 h using the BioTek Cytation 3 Plate Reader or BioTek Synergy H1 Plate Reader (temperature set to 30°C, gain set to 160 for DS40M4 strains).

### Suppressor strain isolation and sequencing

The *Vibrio* parent strain was streaked for isolation on LM plates. In the first round, 4 individual colonies were inoculated separately in M9G shaking at 30°C at 275RPM until turbid. The cultures were then passaged by diluting 1:1,000 into fresh M9G until visible robust growth was seen, which was 12 passages for Δ*qrr1-5* and 17 passages for Δ*luxO*. We sequenced 1 strain for the Δ*qrr1-5* parent strain that grew the most robustly, and we sequenced 3 for the Δ*luxO* parent strain; we report the sequence of one strain in this manuscript.

Sequencing and analysis of suppressor strain genomes was performed by the Indiana University Center for Genomics and Bioinformatics. Two methods were used for Breseq analysis of sequencing reads. Method 1: reads were trimmed using fastp (version 0.20.1) with parameters “-l 17—detect_adapter_for_pe -g -p” [49]. Breseq pipeline 0.36.[50] with bowtie2-2.4. [51] 2 was run with default parameters for mutation calling and annotation by re-querying against the *Vibrio campbellii* strain DS40M4 genome assembly (GenBank accession: GCA_003312585.1). Method 2: reads were adapter trimmed and quality filtered using Trimmomatic 0.38 [52] with the cutoff threshold for average base quality score set at 20 over a window of 3 bases and requiring a read length of at least 20 bp after trimming. Breseq pipeline 0.37.0 [50] with bowtie2-2.4.2 [51] was run with default parameters for mutation calling and annotation by re-querying against the *Vibrio campbellii* strain DS40M4 genome assembly (GenBank accession: GCA_003312585.1). The SRA accession number is SRP482837. The BioProject accession number for these data is PRJNA1063211.

### Competition assays

Monocultures were grown overnight in LM in culture tubes. The next day, 500 μl of cells were pelleted by centrifugation, resuspended in 500 μl M9 medium, spun down again, and resuspended in 500 μl M9, and monocultures were diluted 1:50 in minimal M9 media supplemented with either 20 mM glucose, 0.5% casein, or 0.2% casamino acids and grown at 30°C shaking until ~0.5 OD_600_. Monocultures were adjusted to 0.5 OD_600_ with fresh media and were mixed at the desired population ratios. Cocultures were diluted 1:10,000 and grown at 30°C shaking at 275 RPM. Colony-forming units (cfus/mL) were measured by plating serial dilutions on selective agar media. For competition experiments, where cocultures were maintained in exponential phase, cocultures were diluted to 0.05 OD_600_ at the 16- and 40-h time points.

### RNA extraction and reverse transcriptase quantitative PCR (RT-qPCR)

RNA was extracted and qRT-PCR was performed as previously described in Simpson and colleagues [8] with the exception that the monocultures were grown in M9GC and collected at an ~1 OD_600_ or grown in M9G and collected at an ~0.05 OD_600_ and ~1 OD_600._ Primers in S3 Table.

### LuxR protein purification

Both WT and G37V LuxR from *V*. *campbellii* DS40M4 were overexpressed in *E*. *coli* BL21(DE3) using plasmids pZC003 and pZC004 (S2 Table), respectively, derived from pET28b (Twist Bioscience). Overexpression and purification were performed as described in Newman and van Kessel [17]. The only exceptions being the use of 2.5 ml of 10× FastBreak Cell Lysis Reagent (Promega) to lyse the 1 L cell pellets and the Gel Filtration Buffer (25 mM Tris-HCl (pH 8), 500 mM NaCl, and MilliQ H_2_O) used to elute the proteins. Peak eluted fractions were pooled and analyzed using SDS-PAGE. The pooled fractions were then used in all EMSAs.

### EMSAs

Fluorescently labeled primers were ordered through Integrated DNA Technologies and one labeled primer was utilized in each PCR amplified substrate. EMSAs were performed as described in Newman and van Kessel [17]. The P_*metJ*_ DNA substrate was created by PCR amplifying the 239 bp segment from *V*. *campbellii* DS40M4 gDNA using primers 5′ IR700 Dye ZC011 and ZC012. *P*_*mutS*_ substrate was generated by PCR amplifying a 200 bp segment from *V*. *campbellii* BB120 using primers 5′ IR800 Dye PP378 and PP376. Site H substrates were generated by annealing primers 5′ IR800 Dye JCV369 and JCV620. The gels were imaged on an Odyssey M from LICORbio using the IR700 and IR800 settings.

### Measurements of glucose and DPD

For measurements of glucose, 5 ml cultures were grown overnight in LM at 30°C shaking at 275 RPM. Overnight cultures were washed with M9G and back-diluted 1:100,000 into flasks containing fresh M9G. Cultures were grown shaking at 30°C at 270 RPM. At each sampling, the OD_600_ was recorded and 1.5 ml of culture was spun down at 13,000 RPM. Supernatant (0.4 ml) was filtered (0.45 μm) and glucose was quantified by HPLC equipped with a refractive index detector (Shimadzu) as described in McKinlay and colleagues [53].

For measurements of DPD, supernatant extracts were collected from overnight cultures (OD_600_ > 1.4) grown in 5 ml of M9G. For 250 μl of supernatant, 3 μl of 1 mM OPD was added to derivatize the samples and induce a conformational change of DPD molecules into DPDQ. The samples were analyzed by mass spectrometry as previously described in Lydick and colleagues [54].

## Supporting information

S1 Fig DS40M4 signaling to LuxO. Multiple histidine kinase receptors detect external molecules such as autoinducers that converge to phosphorylate the response regulator LuxO. The lighter colored receptors indicate that they are predicted to be present in DS40M4 but have not been formally tested. Created with BioRender.com.
(TIF)

S2 Fig (A–C) Growth curves for *V*. *campbellii* BB120 strains in LM (A), M9G (B), or M9GC (C) media. (D) Variability in lag phase of HCD-locked DS40M4 mutants grown in M9G medium. Three additional biological replicates of experiments in Fig 1C are shown. (E, F) The difference in cfus/ml between time points 1 and 2 (F) of wild-type and *luxO D47E* DS40M4 strains grown in M9G (F). Error bars show the mean and standard deviation of 5 biological replicates. (G) Growth curves for *V*. *campbellii* DS40M4 strains in M9 medium supplemented with maltose (10 mM). (H–K) Growth curves shown for *V*. *coralliilyticus* (H,J) or *V*. *cholerae* (I, K) grown in M9G (H, I) or M9GC (J, K). (A–E, G–K) The y-axis represents cell density OD_600_. For all panels, the data are from a single experiment that is representative of at least 3 independent biological experiments for each strain under every condition. The data underlying this figure can be found in S6 Data.
(TIF)

S3 Fig (A) Mutations in a Δ*luxO* suppressor mutant strain. (B–E) Growth curves for *V*. *campbellii* DS40M4 strains. The y-axis represents cell density OD_600_. (D) The (+) indicates chromosomal complementation of gene at non-native locus. The inset in panel D is a zoomed-in view of the same data to enable visualization of strains at early time points. (F) Viable cell counts of DS40M4 strains washed and diluted into M9G medium. For panels B–F, the data shown are from a single experiment that is representative of at least 3 independent biological experiments for each strain. The data underlying this figure can be found in S7 Data.
(TIF)

S4 Fig (A) EMSAs with either purified WT LuxR (left) or G37V LuxR (right) with 5′ IR800 (Integrated DNA Technologies) DNA substrate P*luxC* site H (JCV369 and JCV620). Protein concentrations are 0.0005, 0.005, 0.05, 0.5, 5, 50, and 500 nM, compared to no protein control (“-”). This gel is representative of 3 assays performed with 3 individual protein preps. (B) EMSAs with either purified WT LuxR (left) or G37V LuxR (right) with 5′ IR800 (Integrated DNA Technologies) DNA substrate *mutS* (PP376 and PP378). Protein concentrations are 0.0005, 0.005, 0.05, 0.5, 5, 50, and 500 nM, compared to no protein control (“-”). The data underlying this figure can be found in S1 Raw Images.
(TIF)

S5 Fig (A) Growth assay in M9 medium + 0.5% casein (M9 Casein). The y-axis represents cell density OD_600_. (B, C) Wild-type (marked with Tm) and *luxO D61E* (marked with Spec) strains of DS40M4 were inoculated at initial frequencies of 1%–99% wild-type (WT) in M9Casein medium (B) or M9 Casamino acids medium (C). The data in A–C are from a single experiment that is representative of at least 3 independent biological experiments. The data underlying this figure can be found in S8 Data.
(TIF)

S6 Fig (A) Specific glucose consumption rates were determined using r_S_ = Y_XS_ • k, where r_S_ is the specific glucose consumption rate, Y_XS_ is glucose consumed per unit biomass, and k is the exponential growth rate [55,56]. Y_XS_ was determined from the difference between glucose measurements between inoculation and late exponential/early stationary phase. Error bars = SD; *n* = 3. Different letters indicate statistically different values (*p* < 0.05) as determined by ordinary one-way ANOVA with Tukey’s multiple comparison (B) Glucose consumed = (Glucose t_0_—Glucose t_x_) / (Glucose t_0_) × 100%, where t_0_ = the time of inoculation and t_x_ = time after culture growth had ceased. All glucose measurements at t_x_ were below the limit of detection. (C) Replicates of competition experiments with wild-type and *luxO D61E* strains. Wild-type (marked with Tm) and *luxO D61E* (marked with Spec) strains of DS40M4 were inoculated at initial frequencies of 80%, 50%, or 20% wild-type (WT) in M9G medium and grown to near stationary phase at t = 16 h, and then diluted to maintain growth in log-phase. Colony forming units (cfus/mL) were measured at each time point using selective antibiotics for each strain. The total population number was calculated, and the data are represented as the percent of WT cells in the total population of WT and *luxO D61E*. The data underlying this figure can be found in S9 Data.
(TIF)

S1 Table Strains used in this study. (DOCX)

S2 Table Plasmids used in this study. (DOCX)

S3 Table Oligonucleotides used in this study. (DOCX)

S1 Data Fig 1. Growth trends of *V*. *campbellii* quorum sensing mutants differ in the absence of amino acids.(B–D) Growth curves for *V*. *campbellii* DS40M4 strains in LM (B), M9G (C), or M9GC (D) media. The y-axis represents cell density OD_600._ For all panels, the data are from a single experiment that is representative of at least 3 independent biological experiments for each strain under every condition.
(XLSX)

S2 Data Fig 2. Mutation of *metF* or *luxR* restores growth to HCD-locked strains. (B) Culture growth of *V*. *campbellii* DS40M4 strains in M9G medium. (C) Growth rates of strains from panel B are plotted. (D) Growth curves of *V*. *campbellii* DS40M4 strains in M9GC medium. The inset in panels B and D are zoomed-in views of the same data to enable visualization of strains at early time points. (E) Growth rates of strains from panel D are plotted. For panels B and D, the data are from a single experiment that is representative of at least 3 independent biological experiments for each strain under every condition and the y-axis represents cell density OD_600._ For panels C and E, error bars show the mean and standard deviation of at least 3 biological replicates. The slope of the line was derived from the data in exponential phase with at least 5 data points per growth curve. Statistical analyses were performed using the Kruskal–Wallis test (nonparametric) followed by Dunn’s multiple comparisons test (*n =* at least *3*). Different letters above the bars indicate that the pair-wise comparison is significantly different; the same letters above 2 bars indicate no significant differences (*p <* 0.05). There were no significant differences in panel D. Fig 3. LuxR G37V has decreased DNA binding activity. (C) Bioluminescence production (light divided by OD_600_) throughout a growth curve for DS40M4 strains. The data shown are from a single experiment that is representative of at least 3 independent biological experiments for each strain under every condition.
(XLSX)

S3 Data Fig 5. LuxR regulation of methionine biosynthesis genes. (B–G) Relative transcript levels determined by RT-qPCR for cultures grown in M9GC medium (B–D) or M9G medium (E–G). Error bars show the mean and standard deviation of 3 biological replicates. (B–D) Different letters above the bars indicate that the pair-wise comparison is significantly different; the same letters above 2 bars indicate no significant differences (*p <* 0.05). One-way analysis of variance (ANOVA) was performed on log-normalized data (normally distributed, Shapiro–Wilk test; *n* = 3 biological replicates, Tukey’s multiple comparisons test). (E–G) Student’s–test was performed data (normally distributed, Shapiro–Wilk test; *n* = 3 biological replicates; *p* < 0.05, *; *p* < 0.001, ***). (H) Quantification of DPDQ (derivatization of DPD, the precursor to AI-2) via mass spectrometry from supernatants from strains grown in M9G medium and collected at HCD. DPDQ levels were normalized to an internal control (Verapamil) spiked in every sample. The mean of 3 independent biological replicates is shown.
(XLSX)

S4 Data Fig 6. Altering flux between the activated methyl cycle and folate cycle affects culture growth. (A–C) Growth curves for *V*. *campbellii* DS40M4 strains grown in M9G (A, B) or M9GC (C) medium. The y-axes represent cell density OD_600._ The data shown are from a single experiment that is representative of at least 3 independent biological experiments for each strain under every condition. Fig 7. The Qrrs regulate growth of *V*. *campbellii* in minimal medium. (A, B) Growth curves for *V*. *campbellii* DS40M4 strains in M9G medium. The y-axis represents cell density OD_600._ The data shown are from a single experiment that is representative of at least 3 independent biological experiments for each strain under every condition.
(XLSX)

S5 Data Fig 8. Wild-type cells outcompete locked-LCD cells in co-culture in minimal medium. (A) Wild-type (marked with Tm) and *luxO D61E* (marked with Spec) strains of DS40M4 were inoculated at initial frequencies from 1%–99% wild-type (WT) in M9G medium and allowed to grow for 48 h. (B, C) Wild-type (marked with Tm) and *luxO D61E* (marked with Spec, panel B) or Δ*qrr1-5* (marked with Spec, panel C) strains of DS40M4 were inoculated at initial frequencies of 80%, 50%, or 20% wild-type (WT) in (B) M9G medium or (C) M9 Casein medium and grown to near stationary phase at t = 16 h, and then diluted to maintain growth in log-phase. Colony forming units (cfu/mL) were measured at each time point using selective antibiotics for each strain. The total population number was calculated, and the data are represented as the percent of WT cells in the total population of WT and *luxO D61E* or WT and Δ*qrr1-5*. The data are representative of 3 biological experiments.
(XLSX)

S6 Data S2 Fig. (A–C) Growth curves for *V*. *campbellii* BB120 strains in LM (A), M9G (B), or M9GC (C) media. (D) Variability in lag phase of HCD-locked DS40M4 mutants grown in M9G medium. Three additional biological replicates of experiments in Fig 1C are shown. (E, F) The difference in cfus/ml between time points 1 and 2 (F) of wild-type and *luxO D47E* DS40M4 strains grown in M9G (F). Error bars show the mean and standard deviation of 5 biological replicates. (G) Growth curves for *V*. *campbellii* DS40M4 strains in M9 medium supplemented with maltose (10 mM). (H–K) Growth curves shown for *V*. *coralliilyticus* (H, J) or *V*. *cholerae* (I, K) grown in M9G (H, I) or M9GC (J, K). (A–E, G–K) The y-axis represents cell density OD600. For all panels, the data are from a single experiment that is representative of at least 3 independent biological experiments for each strain under every condition.
(XLSX)

S7 Data S3 Fig. (B–E) Growth curves for *V*. *campbellii* DS40M4 strains. The y-axis represents cell density OD600. (D) The (+) indicates chromosomal complementation of gene at non-native locus. The inset in panel D is a zoomed-in view of the same data to enable visualization of strains at early time points. (F) Viable cell counts of DS40M4 strains washed and diluted into M9G medium. For panels B–F, the data shown are from a single experiment that is representative of at least 3 independent biological experiments for each strain.
(XLSX)

S8 Data S5 Fig. (A) Growth assay in M9 medium + 0.5% casein (M9 Casein). The y-axis represents cell density OD600. (B, C) Wild-type (marked with Tm) and *luxO D61E* (marked with Spec) strains of DS40M4 were inoculated at initial frequencies of 1%–99% wild-type (WT) in M9Casein medium (B) or M9 Casamino acids medium (C). The data in A–C are from a single experiment that is representative of at least 3 independent biological experiments.
(XLSX)

S9 Data S6 Fig. (A) Specific glucose consumption rates were determined using r_S_ = Y_XS_ • k, where r_S_ is the specific glucose consumption rate, Y_XS_ is glucose consumed per unit biomass, and k is the exponential growth rate [55,56]. Y_XS_ was determined from the difference between glucose measurements between inoculation and late exponential/early stationary phase. Error bars = SD; *n* = 3. Different letters indicate statistically different values (*p* < 0.05) as determined by ordinary one-way ANOVA with Tukey’s multiple comparison. (B) Glucose consumed = (Glucose t0—Glucose tx) / (Glucose t0) × 100%, where t0 = the time of inoculation and tx = time after culture growth had ceased. All glucose measurements at tx were below the limit of detection. (C) Replicates of competition experiments with wild-type and *luxO D61E* strains. Wild-type (marked with Tm) and *luxO D61E* (marked with Spec) strains of DS40M4 were inoculated at initial frequencies of 80%, 50%, or 20% wild-type (WT) in M9G medium and grown to near stationary phase at t = 16 h, and then diluted to maintain growth in log-phase. Colony forming units (cfus/mL) were measured at each time point using selective antibiotics for each strain. The total population number was calculated, and the data are represented as the percent of WT cells in the total population of WT and *luxO D61E*.
(XLSX)

S1 Raw Images Fig 3. LuxR G37V has decreased DNA-binding activity. (D, E) EMSAs with either purified WT LuxR (D) or G37V LuxR (E) with 5′ IR700 Dye (Integrated DNA Technologies) DNA substrate P*metJ* region (ZC011 and ZC012). Protein concentrations are 0.0005, 0.005, 0.05, 0.5, 5, 50, and 500 nM, compared to no protein control (“-”). S4 Fig. (A) EMSAs with either purified WT LuxR (left) or G37V LuxR (right) with 5′ IR800 (Integrated DNA Technologies) DNA substrate P*luxC* site H (JCV369 and JCV620). Protein concentrations are 0.0005, 0.005, 0.05, 0.5, 5, 50, and 500 nM, compared to no protein control (“-”). This gel is representative of 3 assays performed with 3 individual protein preps. (B) EMSAs with either purified WT LuxR (left) or G37V LuxR (right) with 5′ IR800 (Integrated DNA Technologies) DNA substrate *mutS* (PP376 and PP378). Protein concentrations are 0.0005, 0.005, 0.05, 0.5, 5, 50, and 500 nM, compared to no protein control (“-”).
(PDF)

## Abbreviations

AMC: activated methyl cycle
cfu: colony-forming unit
EMSA: electrophoretic mobility shift assay
HCD: high cell density
HPLC: high-pressure liquid chromatography
LCD: low cell density
LM: lysogeny broth marine
OD: optical density
OPD: orthophenylene damine
QS: quorum sensing
THF: tetrahydrofolate
