# Supplementary figures and images for: Quorum sensing in Vibrio controls carbon metabolism to optimize growth in changing environmental conditions
Source: PLoS Biol. 2024 Nov 11;22(11):e3002891. doi: 10.1371/journal.pbio.3002891 (PMC11581408; doi:10.1371/journal.pbio.3002891)

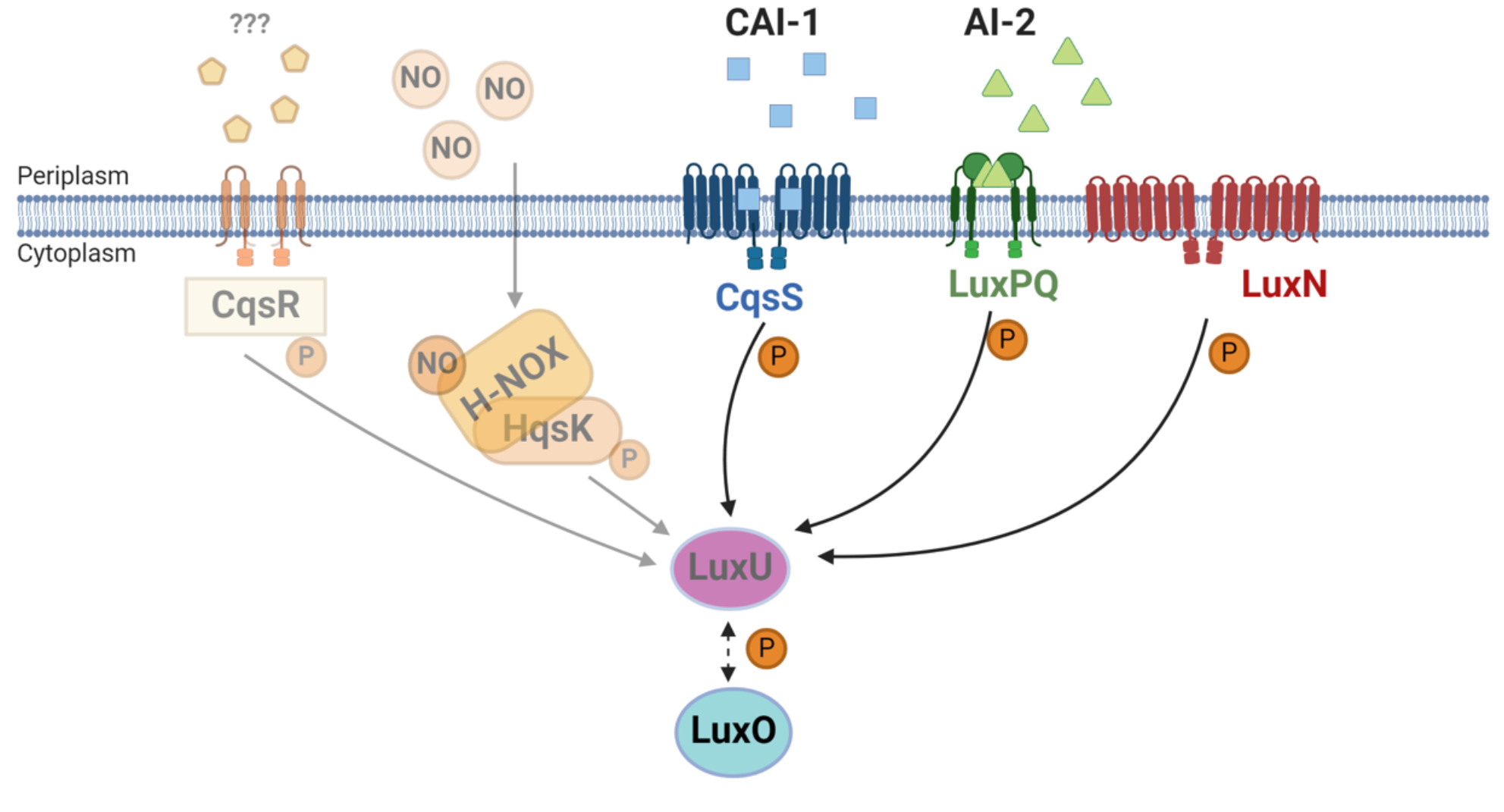

Supplement: S1 Fig — Multiple histidine kinase receptors detect external molecules such as autoinducers that converge to phosphorylate the response regulator LuxO. The lighter colored receptors indicate that they are predicted to be present in DS40M4 but have not been formally tested. Created with BioRender.com. (TIF) [file pbio.3002891.s001.tif]

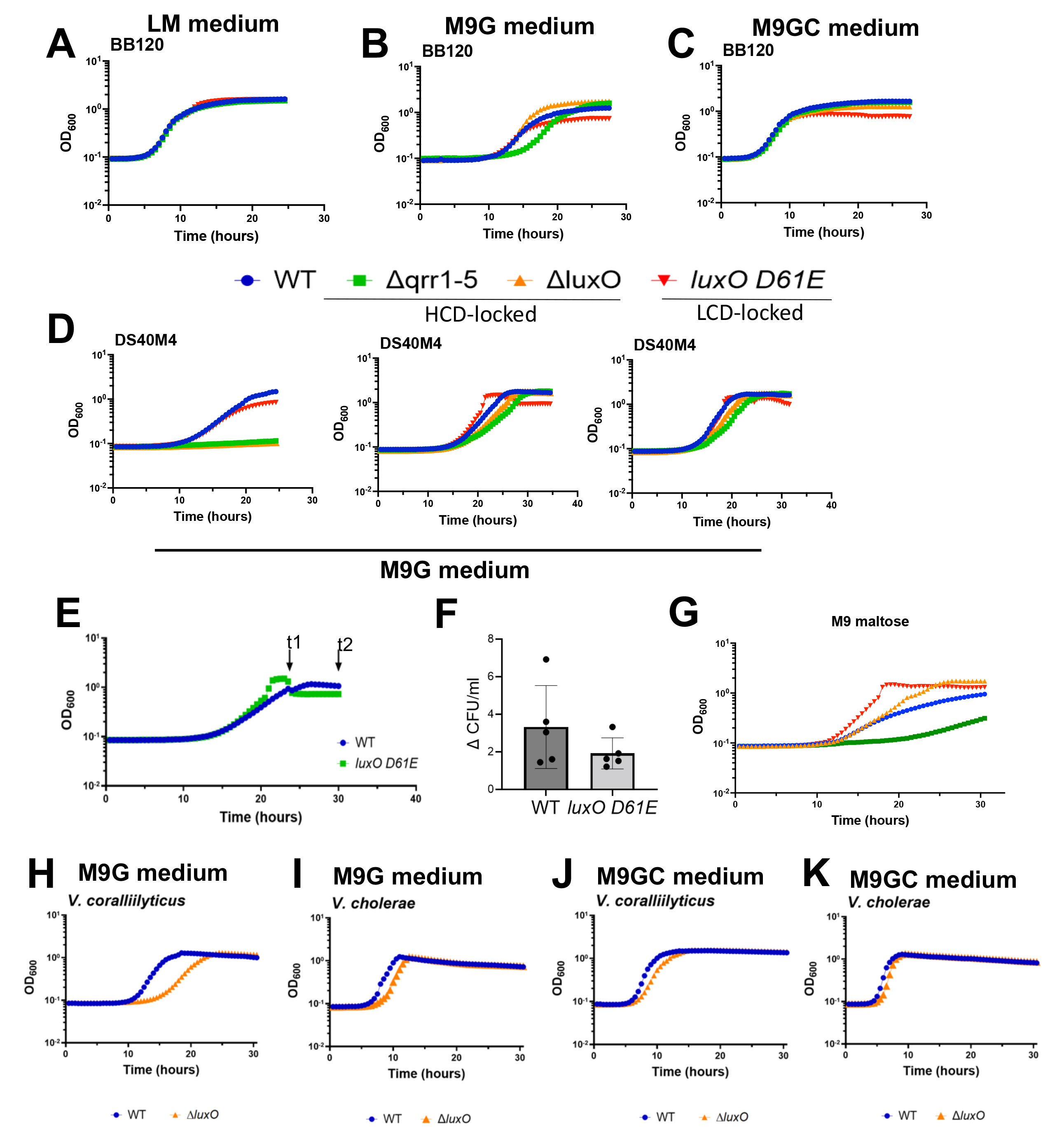

Supplement: S2 Fig — (A–C) Growth curves for V. campbellii BB120 strains in LM (A), M9G (B), or M9GC (C) media. (D) Variability in lag phase of HCD-locked DS40M4 mutants grown in M9G medium. Three additional biological replicates of experiments in Fig 1C are shown. (E, F) The difference in cfus/ml between time points 1 and 2 (F) of wild-type and luxO D47E DS40M4 strains grown in M9G (F). Error bars show the mean and standard deviation of 5 biological replicates. (G) Growth curves for V. campbellii DS40M4 strains in M9 medium supplemented with maltose (10 mM). (H–K) Growth curves shown for V. coralliilyticus (H,J) or V. cholerae (I, K) grown in M9G (H, I) or M9GC (J, K). (A–E, G–K) The y-axis represents cell density OD600. For all panels, the data are from a single experiment that is representative of at least 3 independent biological experiments for each strain under every condition. The data underlying this figure can be found in S6 Data. (TIF) [file pbio.3002891.s002.tif]

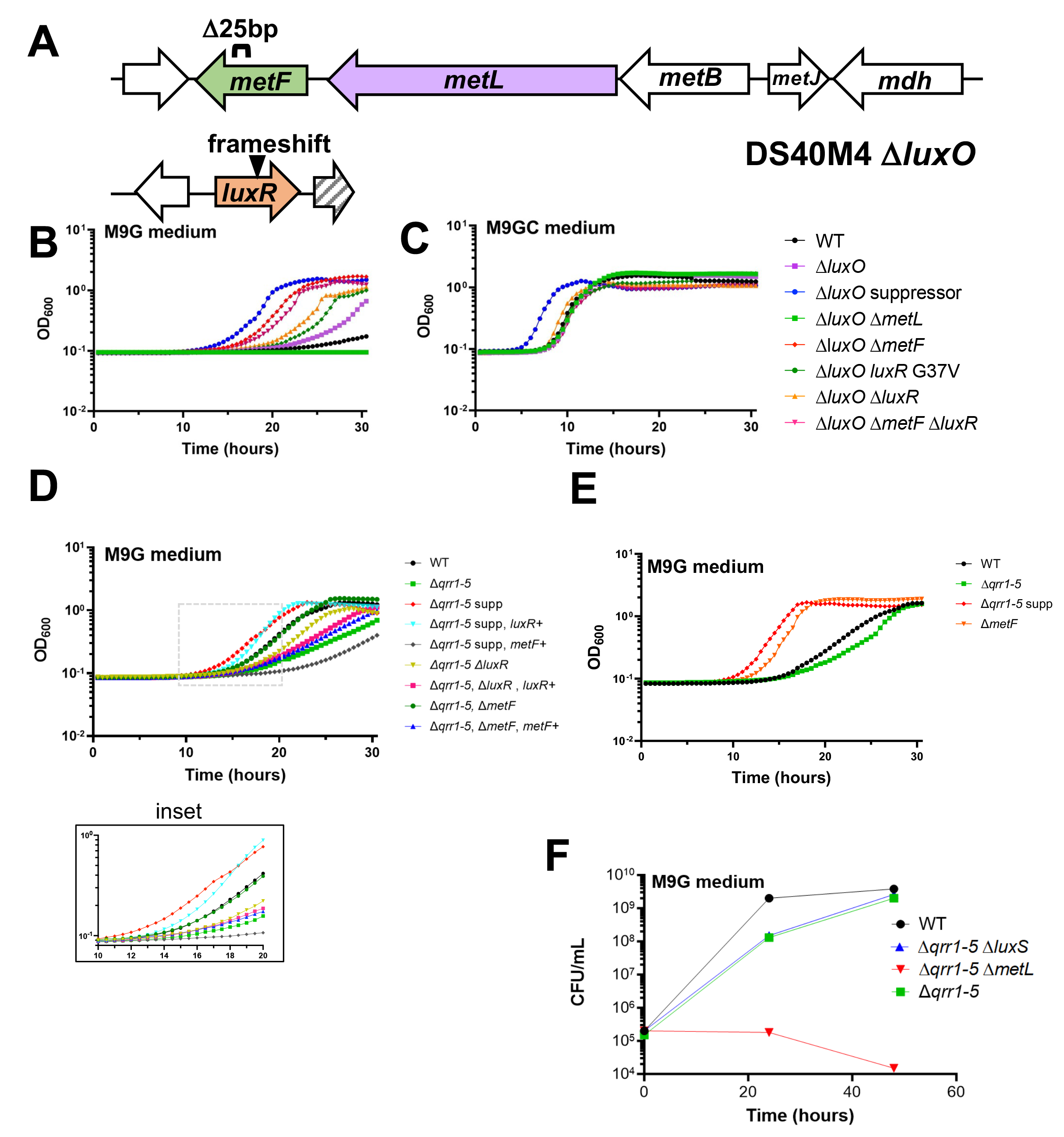

Supplement: S3 Fig — (A) Mutations in a ΔluxO suppressor mutant strain. (B–E) Growth curves for V. campbellii DS40M4 strains. The y-axis represents cell density OD600. (D) The (+) indicates chromosomal complementation of gene at non-native locus. The inset in panel D is a zoomed-in view of the same data to enable visualization of strains at early time points. (F) Viable cell counts of DS40M4 strains washed and diluted into M9G medium. For panels B–F, the data shown are from a single experiment that is representative of at least 3 independent biological experiments for each strain. The data underlying this figure can be found in S7 Data. (TIF) [file pbio.3002891.s003.tif]

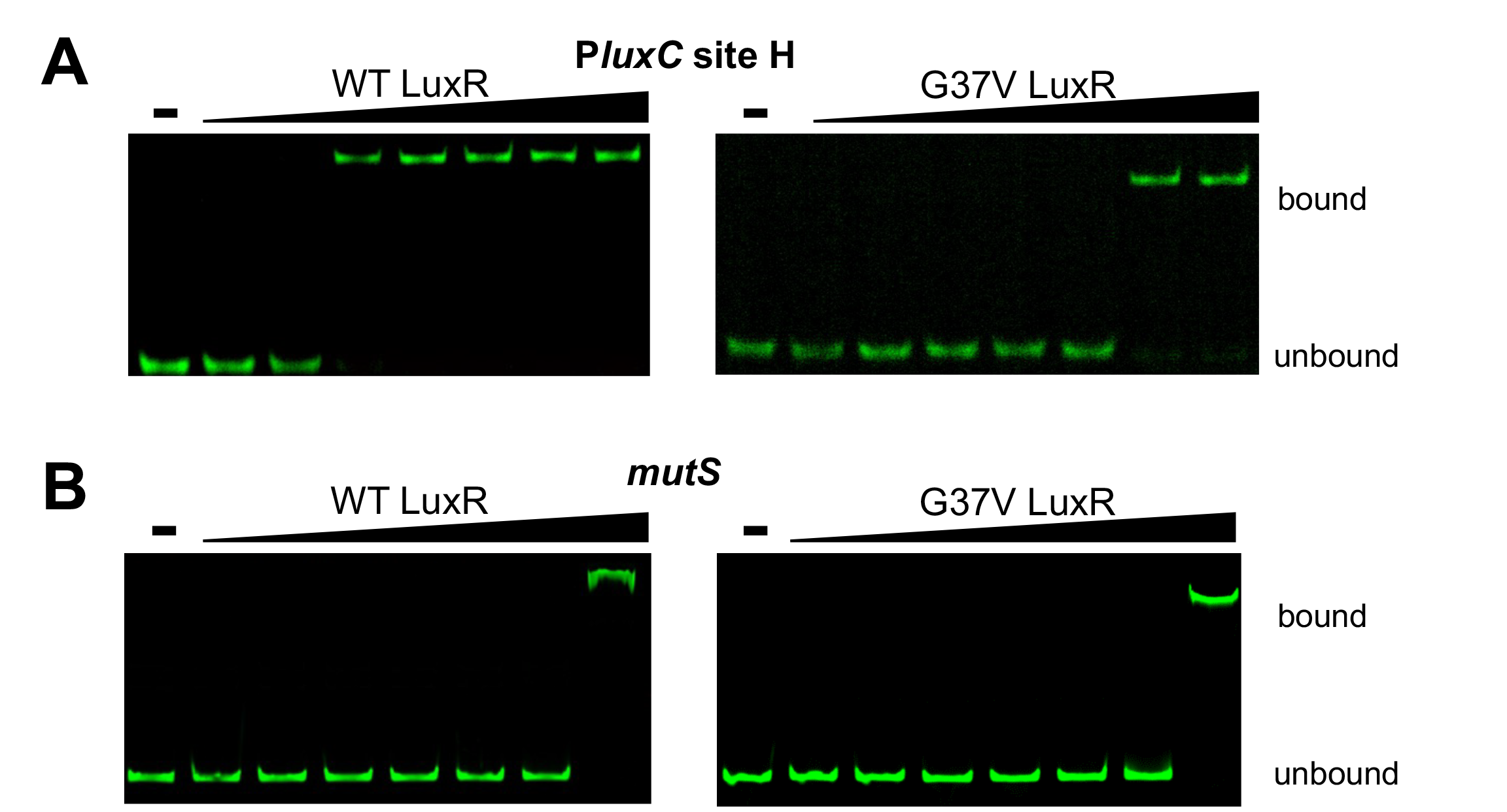

Supplement: S4 Fig — (A) EMSAs with either purified WT LuxR (left) or G37V LuxR (right) with 5′ IR800 (Integrated DNA Technologies) DNA substrate PluxC site H (JCV369 and JCV620). Protein concentrations are 0.0005, 0.005, 0.05, 0.5, 5, 50, and 500 nM, compared to no protein control (“-”). This gel is representative of 3 assays performed with 3 individual protein preps. (B) EMSAs with either purified WT LuxR (left) or G37V LuxR (right) with 5′ IR800 (Integrated DNA Technologies) DNA substrate mutS (PP376 and PP378). Protein concentrations are 0.0005, 0.005, 0.05, 0.5, 5, 50, and 500 nM, compared to no protein control (“-”). The data underlying this figure can be found in S1 Raw Images. (TIF) [file pbio.3002891.s004.tif]

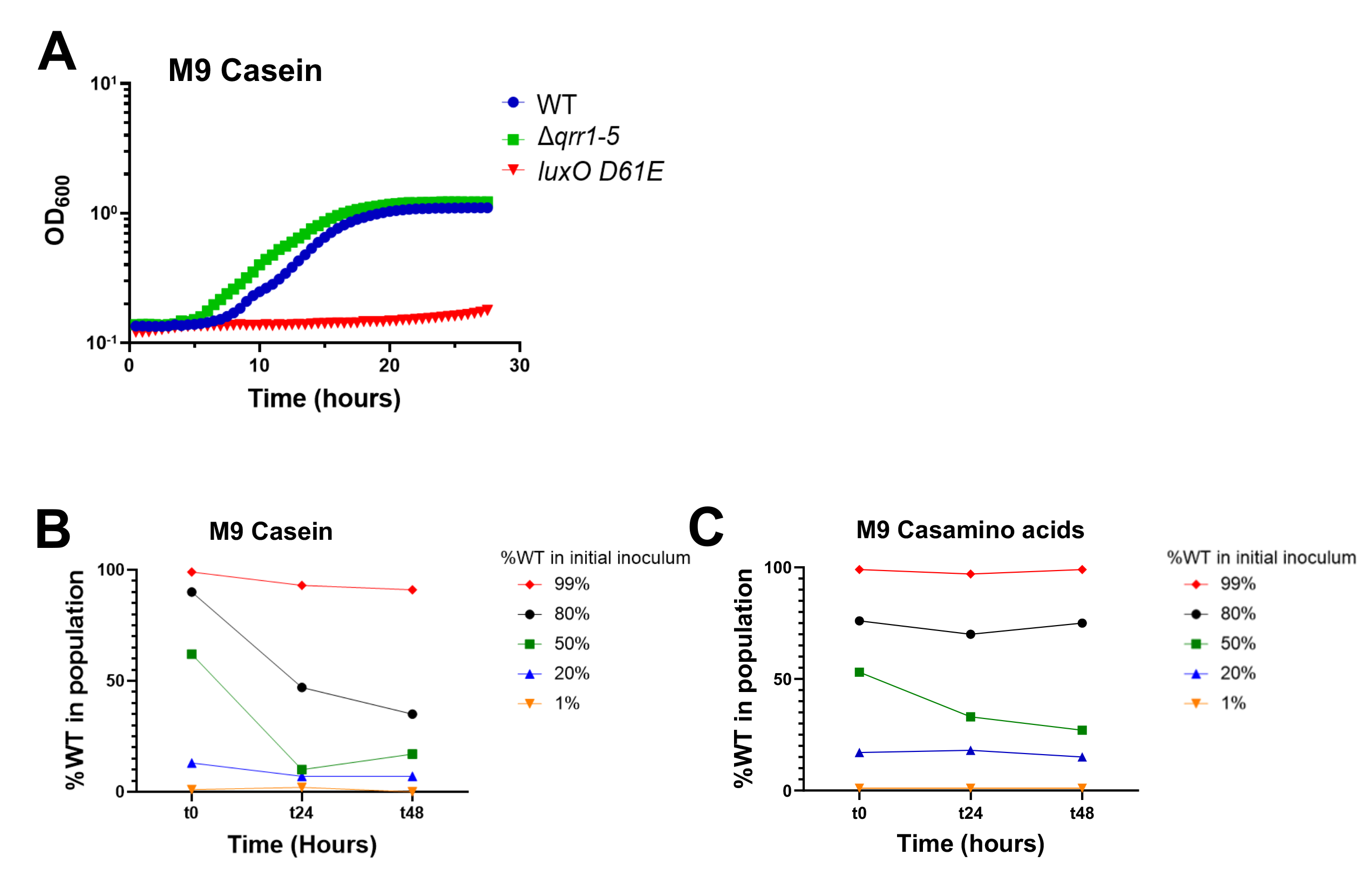

Supplement: S5 Fig — (A) Growth assay in M9 medium + 0.5% casein (M9 Casein). The y-axis represents cell density OD600. (B, C) Wild-type (marked with Tm) and luxO D61E (marked with Spec) strains of DS40M4 were inoculated at initial frequencies of 1%–99% wild-type (WT) in M9Casein medium (B) or M9 Casamino acids medium (C). The data in A–C are from a single experiment that is representative of at least 3 independent biological experiments. The data underlying this figure can be found in S8 Data. (TIF) [file pbio.3002891.s005.tif]

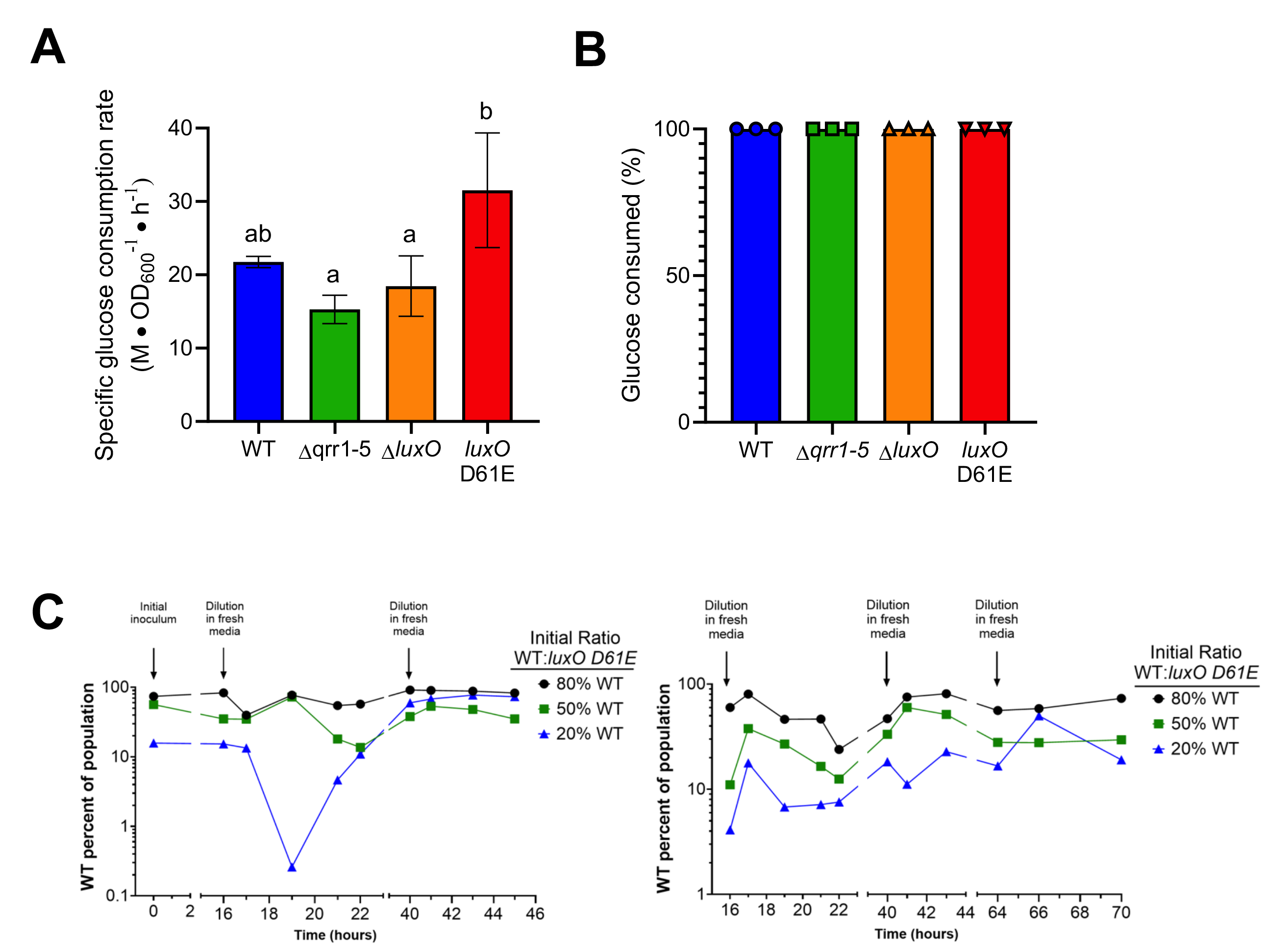

Supplement: S6 Fig — (A) Specific glucose consumption rates were determined using rS = YXS • k, where rS is the specific glucose consumption rate, YXS is glucose consumed per unit biomass, and k is the exponential growth rate [55,56]. YXS was determined from the difference between glucose measurements between inoculation and late exponential/early stationary phase. Error bars = SD; n = 3. Different letters indicate statistically different values (p < 0.05) as determined by ordinary one-way ANOVA with Tukey’s multiple comparison (B) Glucose consumed = (Glucose t0—Glucose tx) / (Glucose t0) × 100%, where t0 = the time of inoculation and tx = time after culture growth had ceased. All glucose measurements at tx were below the limit of detection. (C) Replicates of competition experiments with wild-type and luxO D61E strains. Wild-type (marked with Tm) and luxO D61E (marked with Spec) strains of DS40M4 were inoculated at initial frequencies of 80%, 50%, or 20% wild-type (WT) in M9G medium and grown to near stationary phase at t = 16 h, and then diluted to maintain growth in log-phase. Colony forming units (cfus/mL) were measured at each time point using selective antibiotics for each strain. The total population number was calculated, and the data are represented as the percent of WT cells in the total population of WT and luxO D61E. The data underlying this figure can be found in S9 Data. (TIF) [file pbio.3002891.s006.tif]
