# Supplementary material for: Quorum sensing in Vibrio controls carbon metabolism to optimize growth in changing environmental conditions
Source: PLoS Biol. 2024 Nov 11;22(11):e3002891. doi: 10.1371/journal.pbio.3002891 (PMC11581408; doi:10.1371/journal.pbio.3002891)
Supplement: S1 Table — (DOCX) [file pbio.3002891.s007.docx]

**Table S1.** Strains used in this study.

| Strains | Genotype | Reference |
| --- | --- | --- |
| ***V. campbellii* strains** |  |  |
| BB120 | BB120, wild-type (ATCC BAA-1116) | (1) |
| JAF78 | BB120, Δ*luxO::CM^R^* | (2) |
| JAF548 | BB120, *luxO* D61E*::KanR* | (3) |
| KT282 | BB120, Δ*qrr1-5* | (4) |
| cas107 | DS40M4, Δ*luxB::SpecR* | (5) |
| cas251 | DS40M4, Δ*qrr1-5* | This study |
| cas197 | DS40M4, Δ*luxO* | This study |
| BP60 | DS40M4, *luxO* D61E | (5) |
| cas537 | DS40M4, Δ*qrr1-5* suppressor mutant | This study |
| cas547 | DS40M4, Δ*qrr1-5*, *ΔmetL* | This study |
| cas548 | DS40M4, Δ*qrr1-5*, *ΔmetF* | This study |
| cas549 | DS40M4, Δ*qrr1-5*, *luxR* G37V | This study |
| cas597 | DS40M4, Δ*qrr1-5*, Δ*metF*, *luxR* G37V | This study |
| cas601 | DS40M4, Δ*qrr1-5*, Δ*metF*, Δ*luxR* | This study |
| cas632 | DS40M4, Δ*qrr1-5*, Δ*luxR* | This study |
| cas600 | DS40M4, Δ*luxR*, Δ*metF* | This study |
| cas196 | DS40M4, Δ*luxR* | This study |
| cas279 | DS40M4, Δ*luxS* | This study |
| cas554 | DS40M4, Δ*qrr1-5*, Δ*luxS* | This study |
| cas628 | DS40M4, Δ*qrr1-5*, pMMB-P*tac-luxS* | This study |
| cas602 | DS40M4, Δ*metJ* | This study |
| cas603 | DS40M4, Δ*metJ*, *luxO* D61E | This study |
| cas604 | DS40M4, Δ*metJ*, Δ*qrr1-5* | This study |
| cas622 | DS40M4, Δ*metJ*, Δ*luxR* | This study |
| cas173 | DS40M4, Δ*luxB::TmR* | This study |
| cas625 | DS40M4, Δ*luxO* suppressor mutant | This study |
| cas595 | DS40M4, Δ*luxO*, Δ*metL* | This study |
| cas596 | DS40M4, Δ*luxO*, Δ*metF* | This study |
| cas620 | DS40M4, Δ*luxO*, *luxR* G37V | This study |
| cas630 | DS40M4, Δ*luxO*, Δ*luxR* | This study |
| cas631 | DS40M4, Δ*luxO*, Δ*luxR,* Δ*metF* | This study |
| cas662 | DS40M4, Δ*qrr1-5* suppressor mutant, ∆*luxB::TmR+*m*etF* compliment | This study |
| cas663 | DS40M4, Δ*qrr1-5* suppressor mutant, ∆*luxB::TmR*+*luxR* compliment | This study |
| cas660 | DS40M4, *∆qrr1-5*, *∆MetF*, *∆luxB::SpecR+MetF* compliment | This study |
| cas661 | DS40M4, *∆qrr1-5*, *∆luxR*, *∆luxB::SpecR+LuxR* compliment | This study |
| cas419 | DS40M4, *∆qrr1-5, ∆luxB::Ptac-qrr4 TmR* | This study |
| cas666 | DS40M4, *∆qrr1-5, ∆luxR, ∆luxB::Ptac-qrr4 TmR* | This study |
| cas683 | DS40M4, *∆metE, ∆metH* | This study |
|  |  |  |
| ***V. coralliilyticus* strains** |  |  |
| lab strains Vco001 | OCN008 wild-type | This study |
| lab strains Vco008 | OCN008 ∆*luxO* | This study |
|  |  |  |
| ***V. cholerae strains*** |  |  |
| lab strains Vc010 | E7946 wild-type | (6) |
| lab strains Vc017 | E7946 ∆*luxO*::SpecR | (7) |
|  |  |  |
| ***E. coli* strains** |  |  |
| S17-1ʎpir | Wild-type, mating strain | (8) |
| pZRC009 | BL21(DE3), pZC005 | This study |
| pZRC010 | BL21(DE3), pZC006 | This study |

**References:**

1. Bassler BL, Greenberg EP, Stevens AM. 1997. Cross-species induction of luminescence in the quorum-sensing bacterium Vibrio harveyi. J Bacteriol 179:4043-5.

2. Freeman JA, Bassler BL. 1999. A genetic analysis of the function of LuxO, a two-component response regulator involved in quorum sensing in Vibrio harveyi. Mol Microbiol 31:665-77.

3. Henke, J. and Bassler, B. (2004) Quorum sensing regulates type III secretion in Vibrio harveyi and Vibrio parahaemolyticus. J Bacteriol 186: 3794–3805.

4. Rutherford, S.T. and Bassler, B.L. (2012) Bacterial quorum sensing: Its role in virulence and possibilities for its control. Cold Spring Harb Perspect Med 2: 1–25.

5. Simpson CA, Petersen BD, Haas NW, Geyman LJ, Lee AH, Podicheti R, Pepin R, Brown LC, Rusch DB, Manzella MP, Papenfort K, van Kessel JC. The quorum-sensing systems of Vibrio campbellii DS40M4 and BB120 are genetically and functionally distinct. Environ Microbiol. 2021 Sep;23(9):5412-5432. doi: 10.1111/1462-2920.15602. Epub 2021 Jun 7. PMID: 33998118; PMCID: PMC8458232.

6. Miller VL, DiRita VJ, Mekalanos JJ. Identification of toxS, a regulatory gene whose product enhances toxR-mediated activation of the cholera toxin promoter. J Bacteriol. 1989;171:1288–1293.

7. Walker LM, Haycocks JRJ, Van Kessel JC, Dalia TN, Dalia AB, Grainger DC. A simple mechanism for integration of quorum sensing and cAMP signalling in *Vibrio cholerae*. Elife. 2023 Jul 6;12:RP86699. doi: 10.7554/eLife.86699. PMID: 37410076; PMCID: PMC10328515.

8. de Lorenzo V, Timmis KN. 1994. Analysis and construction of stable phenotypes in gram-negative bacteria with Tn5- and Tn10-derived minitransposons. Methods Enzymol 235:386-405.
