# Supplementary material for: Quorum sensing in Vibrio controls carbon metabolism to optimize growth in changing environmental conditions
Source: PLoS Biol. 2024 Nov 11;22(11):e3002891. doi: 10.1371/journal.pbio.3002891 (PMC11581408; doi:10.1371/journal.pbio.3002891)
Supplement: S2 Table — (DOCX) [file pbio.3002891.s008.docx]

**Table S2.** Plasmids used in this study.

| Strains | Genotype | Reference |
| --- | --- | --- |
| pCS38 | pMMB67EH, P*luxC*-luxCDABE | (1) |
| pCS48 | pMMB67EH, P*tac-luxS* | (1) |
| pZRC009 | BL21(DE3), pET28b-LuxR (DS40M4) | This study (Twist Bioscience) |
| pZRC010 | BL21(DE3), pET28b-LuxR G37V (DS40M4) | This study (Twist Bioscience) |

**Reference:**

1. Simpson CA, Petersen BD, Haas NW, Geyman LJ, Lee AH, Podicheti R, Pepin R, Brown LC, Rusch DB, Manzella MP, Papenfort K, van Kessel JC. The quorum-sensing systems of Vibrio campbellii DS40M4 and BB120 are genetically and functionally distinct. Environ Microbiol. 2021 Sep;23(9):5412-5432. doi: 10.1111/1462-2920.15602. Epub 2021 Jun 7. PMID: 33998118; PMCID: PMC8458232.
