# Supplementary material for: Quorum sensing in Vibrio controls carbon metabolism to optimize growth in changing environmental conditions
Source: PLoS Biol. 2024 Nov 11;22(11):e3002891. doi: 10.1371/journal.pbio.3002891 (PMC11581408; doi:10.1371/journal.pbio.3002891)
Supplement: S3 Table — (DOCX) [file pbio.3002891.s009.docx]

**Table S3.** Oligonucleotides used in this study.

| Name | Sequence | | | Notes |
| --- | --- | --- | --- | --- |
| **qRT-PCR Primers** | | | | |
| CAS0682 | gcacatcgatgccttaaaccag | | | qRT-PCR DS40M4 *metF* F |
| CAS0683 | cgtttcttccatcttctcactgc | | | qRT-PCR DS40M4 *metF* R |
| CAS0684 | cacttacgtaccctcacctcgt | | | qRT-PCR DS40M4 *metR* F |
| CAS0685 | ctttgatttggtgtgaaagggcg | | | qRT-PCR DS40M4 *metR* R |
| CAS0678 | gctgactggaatggcgaatac | | | qRT-PCR DS40M4 *metJ* F |
| CAS0679 | ttaaaacttttagcgggatagagacgg | | | qRT-PCR DS40M4 *metJ* R |
| BP362 | TGGAAATCGCTCTTGAAGTGT | | | qRT-PCR DS40M4 *luxR F* |
| BP363 | TTAAATACCGTCGCAACAGAAAC | | | qRT-PCR DS40M4 *luxR* R |
| CAS0781 | ACCATCACGGTATTCGACCTAC | | | qRT-PCR DS40M4 *luxS F* |
| CAS0782 | GCATAAAGCCTGCGTACAAGTG | | | qRT-PCR DS40M4 *luxS* R |
| BP345 | ATGGCTAAGGGGCAATCTCTAC | | | qRT-PCR DS40M4 *hfq* F |
| BP346 | CTTGCAGTTTGATACCGTTCAC | | | qRT-PCR DS40M4 *hfq* R |
|  |  | | |  |
| **EMSA Primers** | |  |  | |
| JCV369 | | 5’IRD800-tgatgttatttatatttatatcatttaaataa | F P*_luxC_* Site H | |
| JCV620 | | ttatttaaatgatataaatataaataacatca | R P*_luxC_* Site H | |
| ZC011 | | 5’IRD700-atgagtgtatcctttcacggg | F PCR of 239 bp P*_metJ_* | |
| ZC012 | | tcagccattgcgcacc | R PCR of 239 bp P*_metJ_* | |
| PP376 | | CTACGACGATGCTAAACGCG | F PCR of 200 bp *mutS* open reading frame | |
| PP378 | | 5’IRD800-CAACCGGGCCTTTACTTGTC | R PCR of 200 bp *mutS* open reading frame | |
