# Supplementary material for: Quorum sensing in Vibrio controls carbon metabolism to optimize growth in changing environmental conditions
Source: PLoS Biol. 2024 Nov 11;22(11):e3002891. doi: 10.1371/journal.pbio.3002891 (PMC11581408; doi:10.1371/journal.pbio.3002891)
Supplement: S1 Raw Images — Fig 3. LuxR G37V has decreased DNA-binding activity. (D, E) EMSAs with either purified WT LuxR (D) or G37V LuxR (E) with 5′ IR700 Dye (Integrated DNA Technologies) DNA substrate PmetJ region (ZC011 and ZC012). Protein concentrations are 0.0005, 0.005, 0.05, 0.5, 5, 50, and 500 nM, compared to no protein control (“-”). S4 Fig. (A) EMSAs with either purified WT LuxR (left) or G37V LuxR (right) with 5′ IR800 (Integrated DNA Technologies) DNA substrate PluxC site H (JCV369 and JCV620). Protein concentrations are 0.0005, 0.005, 0.05, 0.5, 5, 50, and 500 nM, compared to no protein control (“-”). This gel is representative of 3 assays performed with 3 individual protein preps. (B) EMSAs with either purified WT LuxR (left) or G37V LuxR (right) with 5′ IR800 (Integrated DNA Technologies) DNA substrate mutS (PP376 and PP378). Protein concentrations are 0.0005, 0.005, 0.05, 0.5, 5, 50, and 500 nM, compared to no protein control (“-”). (PDF) [file pbio.3002891.s019.pdf]

S1_raw_images

A

Representative in Figure S4B

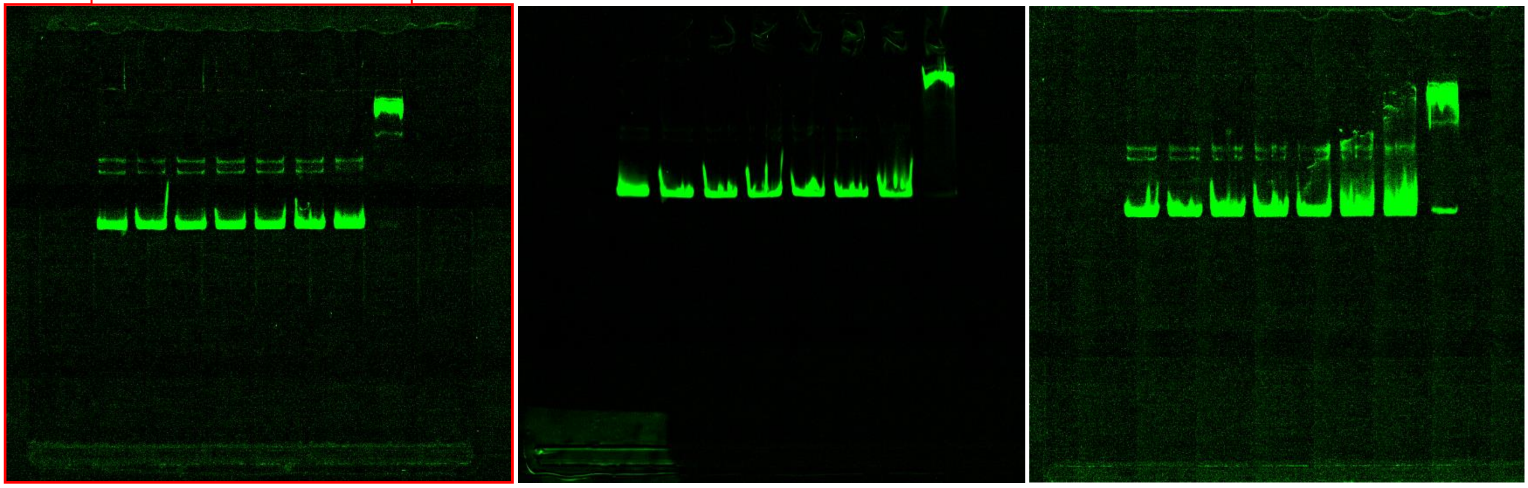

Np .0005 .005 .05 .5 5 50 500 nM   Np .0005 .005 .05 .5 5 50 500 nM   Np .0005 .005 .05 .5 5 50 500 nM

Representative in Figure 3D

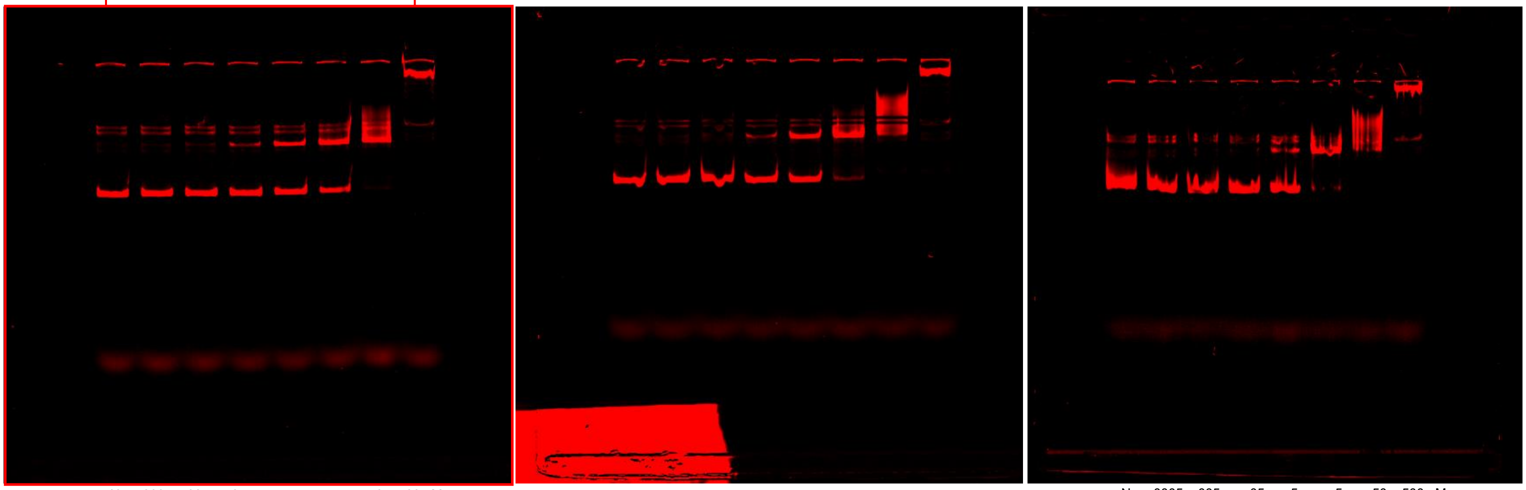

Np .0005 .005 .05 .5 5 50 500 nM   Np .0005 .005 .05 .5 5 50 500 nM   Np .0005 .005 .05 .5 5 50 500 nM

Representative in Figure S4A

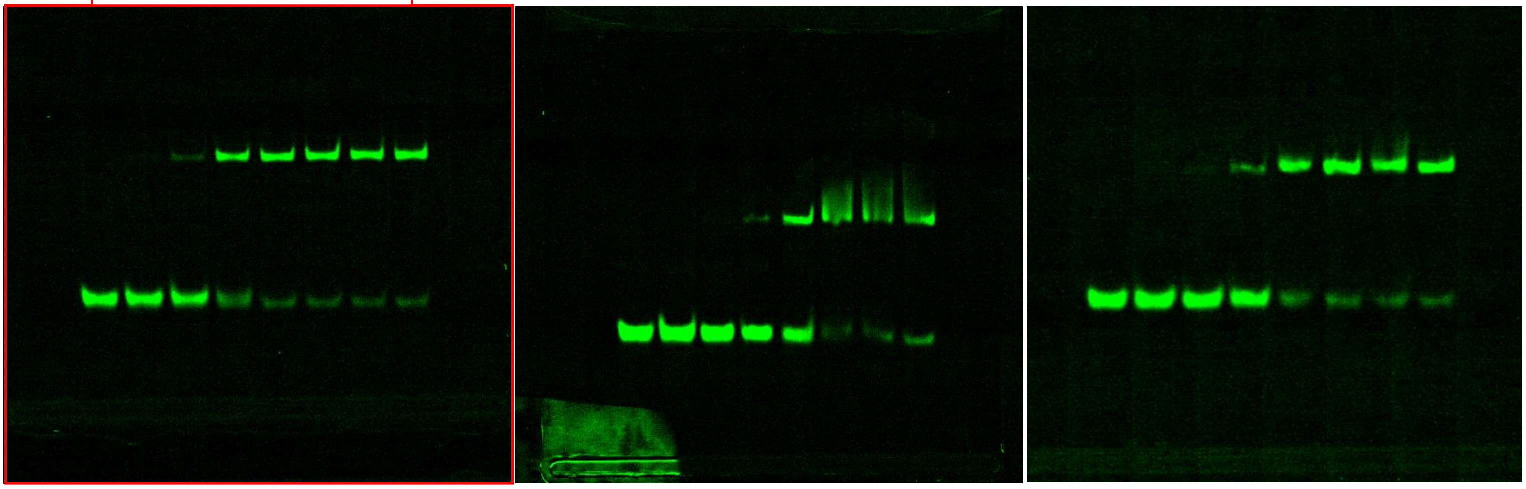

Np .0005 .005 .05 .5 5 50 500 nM   Np .0005 .005 .05 .5 5 50 500 nM   Np .0005 .005 .05 .5 5 50 500 nM

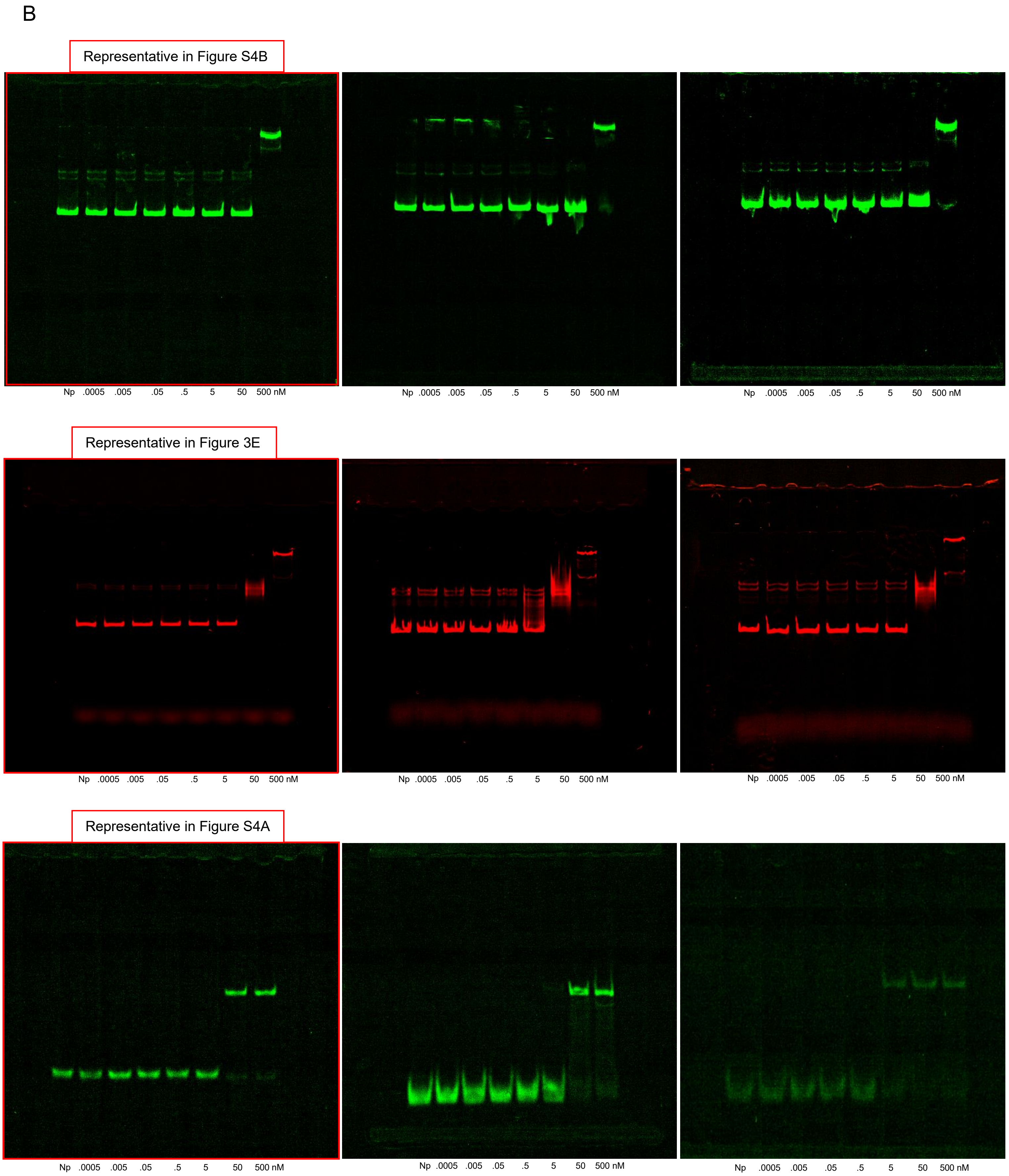

Supplemental Figure 10_Data: Raw EMSAs with either purified WT LuxR (A) or G37V LuxR (B) with 5' IR700 ® Dye (Integrated DNA Technologies) DNA substrate P_{mutS} (PP376 and PP378), P_{metJ} (ZC011 and ZC012), and Site H from $P_{luxcdabe}$ (JCV369 and JCV620), respectively from top to bottom at 1 nM DNA. Protein concentrations are 0.0005, 0.005, 0.05, .5, 5, 50, and 500 nM, compared to no protein control (Np).
